# Effects of blue light and UV-B radiation during growth on photochemical yields during exposure to bright sunlight

**DOI:** 10.1186/s12870-026-08455-4

**Published:** 2026-03-06

**Authors:** Marieke Trasser, T. Matthew Robson, Maxime Durand

**Affiliations:** 1 Cold Spring Harbor Laboratory, 1 Bungtown Rd, Cold Spring Harbor, NY 11724 USA; 2 Organismal and Evolutionary Biology Research Programme (OEB), Viikki Plant Science Centre (ViPS), Faculty of Biological and Environmental Sciences, University of Helsinki, P.O. Box 65, 00014, Helsinki, Finland; 3 UK National School of Forestry, University of Cumbria, Ambleside, UK; 4 Laboratoire Ecologie Société et Evolution, UMR8079, Université Paris-Sud, Orsay, F- 91405 France; 5 CNRS, Orsay, F-91405 France

**Keywords:** Photobiology, Spectral composition, Phenolic compounds, Photosynthesis, Cross tolerance, Plant stress, Photoreceptors, UV radiation, Flavonols, Anthocyanins, Photoprotection, UVR8, Cryptochromes, Phototropins

## Abstract

**Background:**

Many plant responses important for photoprotection in high light environments are mediated by photoreceptors for blue and UV radiation. Perception and response to these shortwave regions at relatively low irradiances may function as a means of priming leaves for the transition to high irradiance. We tested the roles of phototropin (PHOT), cryptochrome (CRY) and ultraviolet-resistance locus 8 (UVR8) photoreceptors in facilitating acclimation to an increase in solar radiation.

**Results:**

*Arabidopsis thaliana* plants deficient in these photoreceptors were grown in a controlled environment under spectral irradiance treatments with or without blue light for 35 days, followed by additional UV-B radiation for 9 days. All plants were then transferred outdoors. Acute high irradiance outdoors caused an immediate drop in photosynthetic yield across genotypes, followed by a midday decline that was less pronounced in wild-type plants grown with blue light and UV-B. Plants grown with blue light but no UV-B were less affected than those with UV-B but no blue. Plants deficient in both UVR8 and CRY performed worst, with CRY having a stronger effect on photoinhibition. Epidermal anthocyanin and flavonol indices were strongly negatively correlated. This relationship was particularly tight in plants from treatments containing blue light and it followed a genotype-specific pattern; whereby genotypes showing the weakest flavonols accumulation (*cry*_*1*_*cry*_*2*_*uvr*_*8-2*_ and *cry*_*1*_*cry*_*2*_) also had the highest anthocyanins.

**Conclusion:**

This pattern suggests metabolic compensation through anthocyanins for the relative lack of flavonol induction. Nevertheless, this compensation mechanism was insufficient to allow plants lacking CRY to maintain photosynthetic function when transferred to outdoor conditions.

**Supplementary Information:**

The online version contains supplementary material available at 10.1186/s12870-026-08455-4.

## Introduction

In nature, the relative proportions of light from different regions of the solar spectrum differ with the environmental conditions, and these differences in spectral composition induce specific plant responses. The classic example of this is canopy shade, which changes not only the ratio of direct to diffuse radiation received but also decreases the red: far-red ratio, inducing the shade avoidance syndrome in many species [1]. Blue light is depleted under plant shade [2] compared with full sunlight; also playing a role in the shade avoidance syndrome [1]. Thus, we start to see that the spectral composition of sunlight changes in a complex manner according to the environment. These changes hold useful information for plants, which have evolved particular responses to differences in the amount of light they receive and to the spectral composition of that light.

Among the short wavelengths at ground level, blue light and ultraviolet (UV) radiation may be used by plants to garner information about their environment. Responses to these spectral regions are coordinated by interacting photoreceptors [1, 3]. Blue light and the ratio of blue: green light are smaller in plant shade. These spectral changes are detected by cryptochromes and phototropins which regulate photosynthesis, stomatal conductance and photomorphogenesis [4]. In addition to blue light, these photoreceptors are also activated by the longer wavelength portion of UV radiation (UV-LW: 350–400 nm) which may further alert plants to the need for enhanced photoprotection, a process activated to prevent damage from excessive light and UV absorption either by dissipating surplus energy (photoinhibition) or shielding photosynthetic components [5, 6]. Finally, the photoreceptor UVR8 (UV resistance locus 8) predominately detects and mediates responses to shortwave UV radiation (UV-SW: 290–350 nm at ground level) [1, 7]. Although all wavebands follow diurnal and seasonal patterns; shortwave UV radiation exhibits stronger variability than longer wavelengths [8], due to Rayleigh scattering, which enriches the UV-B portion of diffuse radiation that comprises shade [9]. As a result, both the absolute amount of UV radiation and the UV: PAR ratio differ between open and shaded environments, and the UV: PAR ratio can further vary among different types of shade. Hence, possession of a specific photoreceptor in UVR8 may be useful allowing plants to respond to UV-SW-specific patterns of irradiance which may require increased photoprotection or screening [10, 11].

A common response to exposure to UV-B radiation [12] and blue light [13, 14], is the accumulation of flavonol glycosides in the adaxial epidermis. By shielding the interior of the leaf from UV radiation [15–17], these compounds reduce damage to the photosynthetic apparatus, with photosystem II (PSII) being particularly vulnerable to UV-B radiation [18, 19]. Some plant species have been found to adjust their epidermal UV screening diurnally to match daily cycles of solar irradiance [8, 20]. Cryptochromes, mediating responses to blue light and UV-A radiation and UVR8 mediating responses to UV-B, contribute to flavonol accumulation [21, 22]. In fact, close interplay between cryptochromes and UVR8 allows fine-tuning of photoprotective responses to different light environments [7, 21]. UV-B radiation also impacts leaf chlorophyll content. It has been reported that moderate supplemental UV-B radiation drives up to 52% more chlorophyll accumulation compared to plants grown under attenuated UV-B radiation [23]. This response may allow higher photosynthetic capacity under high light [23]. However, high UV-B irradiance can indirectly cause damaging oxidative stress to PSII leading to a loss of photosynthetic capacity [24, 25]. How acclimatation to blue light and UV radiation allows plants to adapt to changes in their light environment during development remains poorly understood.

Although high irradiances of UV and photosynthetically active (PAR, 400–700 nm) radiation can cause photoinhibition, defined as a reduction in photosynthetic capacity due to excess light absorption, and damage to PSII, moderate doses of blue light and UV-B radiation act as regulatory signals, triggering photoreceptor-mediated processes, including photoprotection by epidermal flavonols [12, 26–28]. Photoprotective responses to blue light and UV-B radiation under controlled conditions have been well described, but most studies have relied on plants grown under steady light and temperature conditions in growth rooms or chambers, and subsequently exposed to the spectral regions of interest only for a relatively short period of time [11, 16, 29]. While this approach allows the mechanisms of response to specific regions of light to be described, it also simplifies natural conditions where irradiance and spectral composition continuously fluctuate along with other environmental factors [30]. Long term acclimation to growth under a given spectral composition should be expected to produce a phenotype that is particular to those conditions, and to respond differently to a change in environmental conditions according to the spectral composition received during growth. For instance, priming through growth under a spectrum including moderate doses of blue light or UV-B radiation might be expected to prime the response of a mature plant for acute exposure to high irradiances of blue light or UV-B radiation compared with a plant grown under standard constant growth lights (containing little blue and no UV radiation) in controlled conditions [31–33].

We performed a series of experiments on *Arabidopsis thaliana* wild-type (WT) and photoreceptor mutants deficient in their responses to blue light and UV radiation, to test how growth under blue light and UV-B radiation can prime acclimation responses of mature plants for exposure to solar irradiance when transferred into sunlight. By tracking a time course in photoprotection and chlorophyll fluorescence, we could identify which of the known blue-and-UV photoreceptors are important mediators for these responses. Our hypotheses were:

1. That growth under blue light, UV-B radiation, or both these treatments, would increase epidermal flavonol accumulation, leading to enhanced photoprotection that would reduce photoinhibition when subsequently exposed to sunlight.
2. That the absence of functional cryptochromes and UVR8 photoreceptors would reduce the effectiveness of this response to blue light and UV-B radiation respectively, but that the absence of both these photoreceptors would cause the largest reduction in these responses.
3. That as PHOT1 promotes a cry-mediated response to high solar irradiance, PHOT1 would contribute to the maintenance of photosynthetic yield.

We also aimed to examine the relationships between optical measurements of epidermal flavonols and anthocyanins, and leaf chlorophyll, to consider how the accumulation of these pigments contributes to the maintenance of high photosynthetic yield of PSII.

## Results

### Accumulation of flavonols, anthocyanins and chlorophyll during growth under blue light and UV-B radiation treatments and on subsequent exposure to sunlight

 Relative pigment index measured from the adaxial leaf side of both flavonols and anthocyanins differed according to genotype (*P* < 0.001) and were significantly affected by the growing conditions, and by the day after germination (DAG) before (39 & 42 DAG) and after transfer to high lightoutdoors (45 DAG) (*P* < 2.2e-16). Of these effects, light treatment during growth (presence/absence of UV and/or blue light) had the weakest significance on all adaxial leaf pigment index (*P* ≤ 0.038), while the interactions between light treatment and genotype had the strongest statistical significance (*P* < 0.001). Since the three-way interactions were never significant for any of the three pigments analysed, our description of the results concentrates on the significant two-way interactions.

#### Relative adaxial flavonols leaf indexes

**Fig. 1:**
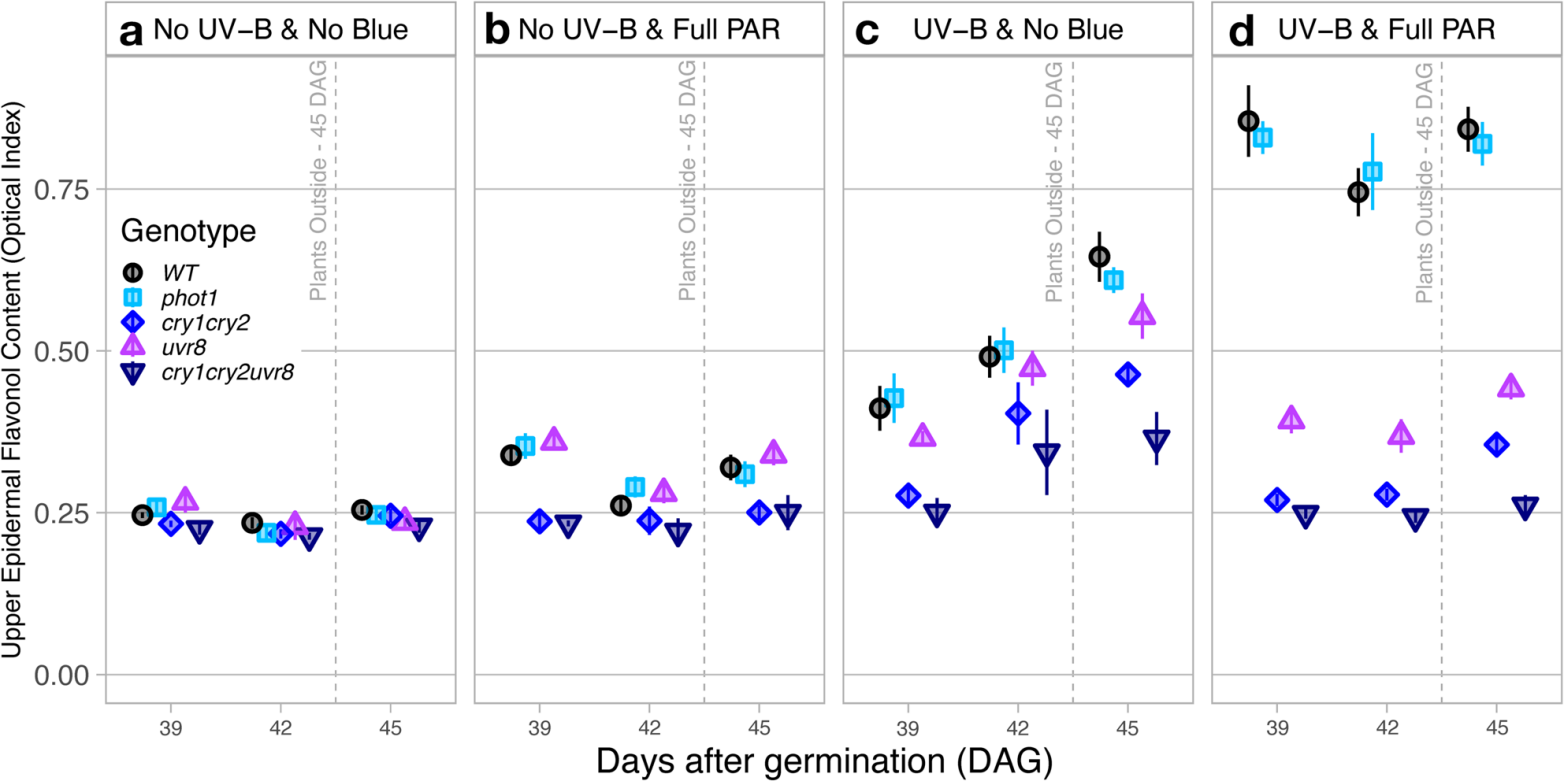
Epidermal flavonols measured optically from the adaxial leaf side - Comparison of flavonols from the adaxial side obtained by Dualex in Arabidopsis wild-type and photoreceptor mutants growing under (**a**) No UV-B & No Blue, (**b**) No UV-B & Full PAR, (**c**) UV-B & No Blue and (**d**) UV-B & Full PAR and to natural sunlight exposure outdoors at midday. Plants were grown under Full PAR or No-Blue conditions from 11 to 35 days after germination, then split into two groups for an additional 5 h daily UV-B treatment over 9 days, with high-light stress applied on day 45. Values shown represent the mean flavonol index ± standard error (SE), calculated from plants grown in three replicate compartments for each treatment. Statistical analyses were performed using ANOVA; full tables are provided in Supplementary Tables S1 and S2

Transfer to high solar irradiance outdoors affected measurements of adaxial flavonol leaf index differently across the genotypes (comparison between 39 DAG and 45 DAG – DAG|Genotype *P* ≤ 0.004, Fig. 1a, b, c, d). Nevertheless, the trends between genotypes and light treatments during growth remained consistent between these measurement days, with generally the highest relative adaxial leaf index measured in WT and *phot1* plants, followed by *uvr*_*8-2*_; while *cry*_*1*_*cry*_*2*_ and *cry*_*1*_*cry*_*2*_*uvr*_*8-2*_ displayed the lowest flavonol index throughout the experiment (Fig. 1a, b, c, d).

Regarding treatment effects, No-UV-B & Full-PAR treatment had no statistically significant effect on the flavonol index in all genotypes compared to No-UV-B & No-Blue (*P* ≥ 0.063, Fig. 1a & b). In contrast, UV-B & No-Blue increased the flavonol index on average 48.5% in WT, 52.3% in *phot1*, 47.1% in *uvr*_*8-2*_ and 38.4% in *cry*_*1*_*cry*_*2*_ compared to No UV-B & No Blue (*P* ≤ 0.001) but had no significant effect on the flavonol index in *cry*_*1*_*cry*_*2*_*uvr*_*8-2*_ (*P* = 0.075, Fig. 1c). Adaxial flavonol leaf index in WT and *phot1* grown in UV-B & Full PAR accumulated on average 37.2% and 29.6% more flavonols respectively (Fig. 1d) than in UV-B & No-Blue (Fig. 1c), and 63.4% and 61.4% more than in No-UV-B & No-Blue (*P* < 0.001, Fig. 1a). Interestingly, *uvr*_*8-2*_ and *cry*_*1*_*cry*_*2*_ grown in UV-B & No-Blue accumulated on average 34.8% and 37.1% more flavonols in the adaxial leaf respectively (Fig. 1c) than in any conditions without UV-B radiation (*P* ≤ 0.002), and *cry*_*1*_*cry*_*2*_*uvr*_*8-2*_ was only marginally affected (31.0%, *P* ≥ 0.075) (Fig. 1a & b).

Treatment effects on flavonols were also different depending on genotype. Adaxial measurements of flavonols on WT, *phot1*, *uvr*_*8-2*_, *cry*_*1*_*cry*_*2*_ and *cry*_*1*_*cry*_*2*_*uvr*_*8-2*_ leaves were similar in No-UV-B & No-Blue conditions (Genotype|Treatment; *P* = 0.950, Fig. 1a), indicating a baseline flavonol index independent of acclimation during growth to blue light or UV-B radiation. In No-UV-B & Full-PAR, only *uvr*_*8-2*_ accumulated 30.7% more adaxial leaf flavonols than *cry*_*1*_*cry*_*2*_*uvr*_*8-2*_ (Genotype|Treatment pairwise; *P* = 0.047) while the relative adaxial flavonol index did not significantly differ with and among other genotypes *(P* ≥ 0.084) (Fig. 1b). Leaves of WT, *phot1* and *uvr*_*8-2*_ showed on average 22.6% higher measurements of adaxial flavonol indexes than *cry*_*1*_*cry*_*2*_ and *cry*_*1*_*cry*_*2*_*uvr*_*8-2*_ (*P* ≤ 0.038) (Fig. 1b). However, WT, *phot1* and *uvr*_*8-2*_ displayed on average similar adaxial flavonol indexes in UV-B & No-Blue conditions (*p* ≥ 0.287), as well as *cry*_*1*_*cry*_*2*_ and *cry*_*1*_*cry*_*2*_*uvr*_*8-2*_ (*P* = 0.171, Fig. 1c). When acclimated to both UV-B & full PAR, adaxial leaf flavonol index was the highest and similar in WT and *phot1* (*p* = 0.341), the lowest and similar in *cry*_*1*_*cry*_*2*_ and *cry*_*1*_*cry*_*2*_*uvr*_*8-2*_ (*P* = 0.143, Fig. 1d). *uvr*_*8-2*_ accumulated on average 48.1% lower adaxial flavonol measurments index than WT and *phot1* (*P* ≤ 0.001), but it was 25.9% higher than in *cry*_*1*_*cry*_*2*_ and *cry*_*1*_*cry*_*2*_*uvr*_*8-2*_ (*P* < 0.025) (Fig. 1d).

#### Relative adaxial anthocyanins leaf index

**Fig. 2:**
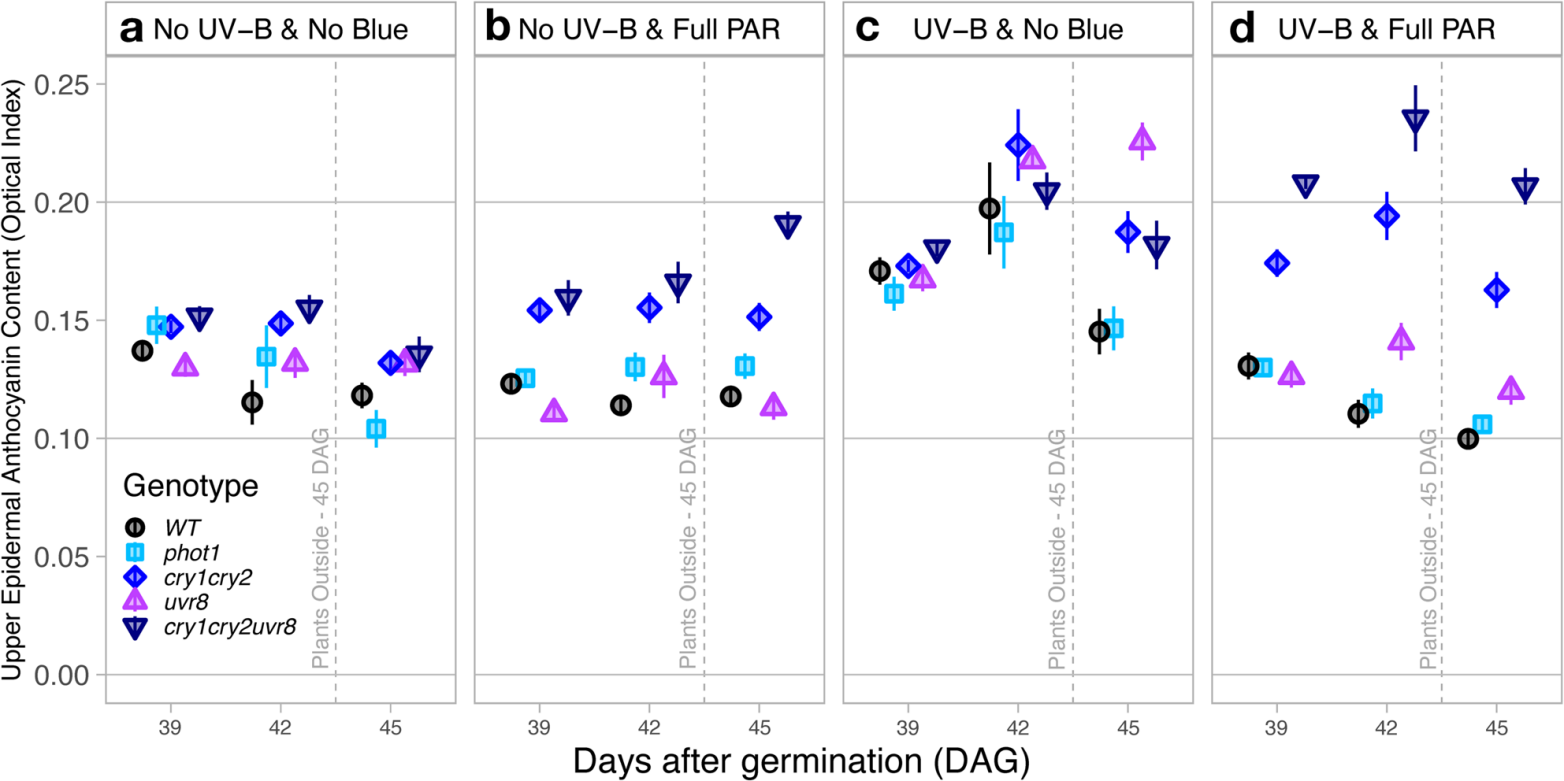
Epidermal anthocyanins measured optically from the adaxial leaf side - Comparison of anthocyanins from the adaxial side obtained by Dualex in Arabidopsis wild-type and photoreceptor mutants growing under (**a**) No UV-B & No Blue, (**b**) No UV-B & Full PAR, (**c**) UV-B & No Blue and (**d**) UV-B & Full PAR and to natural sunlight exposure outdoors at midday. Plants were grown under Full PAR or No-Blue conditions from 11 to 35 days after germination, then split into two groups for an additional 5 h daily UV-B treatment over 9 days, with high-light stress applied on day 45. Values shown represent the mean anthocyanin index ± standard error (SE), calculated from plants grown in three replicate compartments for each treatment. Statistical analyses were performed using ANOVA; full tables are provided in Supplementary Tables S1 and S3

Although the relative anthocyanin index measured from the adaxial leaf side fluctuated over DAG, there was no discernable trend among genotypes between DAG before (39 DAG and 42 DAG) or after sunlight exposure (45 DAG). Generally, anthocyanin index was similar between WT and *phot1* (Genotype|DAG: *P* ≥ 0.389), but their index diverged over time from the other genotypes (Fig. 2a, b, c, d).

Regarding the treatment effect, growth in full PAR did not influence the anthocyanin index compared to conditions without blue light in absence of UV-B in any genotypes (*P* ≥ 0.096, Fig. 2a & b). Anthocyanin content was 20.6% higher on average in all genotypes in UV-B & No Blue than in No UV-B & No Blue conditions (*P* ≤ 0.041, Fig. 2a & c). Although the treatment with full PAR alone (Fig. 2b) did not impact anthocyanin index compared to treatments without full PAR (Fig. 2a), in conditions with combination of UV-B & full PAR anthocyanin measurements were on average 28.5%, 21.9% and 32.8% lower in WT, *phot1* and *uvr*_*8-2*_ respectively (Fig. 2d), compared to those in UV-B & No Blue treatment (*P* ≤ 0.021, Fig. 2c). UV-B & full PAR treatment did statistically significantly affect the relative adaxial anthocyanin leaf index in *cry*_*1*_*cry*_*2*_ and *cry*_*1*_*cry*_*2*_*uvr*_*8-2*_ (Fig. 2d) compared to No UV-B & No Blue (*P* ≤ 0.016) (Fig. 2a).

Treatment effects on anthocyanins were also different depending on genotype. In No UV-B & No Blue treatments, WT, *phot1* and *uvr*_*8-2*_ accumulated similar relative adaxial anthocyanin leaf indexes (*P* ≥ 0.169, Fig. 2a), while were also similar to each other *cry*_*1*_*cry*_*2*_ and *cry*_*1*_*cry*_*2*_*uvr*_*8-2*_ (*P* = 0.788). Comparing these two groups of mutants which behaved similarly, adaxial anthocyanins leaf index in WT, *phot1* and *uvr*_*8-2*_ were 10.4% lower than in *cry*_*1*_*cry*_*2*_ and *cry*_*1*_*cry*_*2*_*uvr*_*8-2*_ (*P* ≤ 0.049, Fig. 2a). In No UV-B & full PAR, relative adaxial anthocyanin accumulation in *cry*_*1*_*cry*_*2*_ and *cry*_*1*_*cry*_*2*_*uvr*_*8-2*_ were on average 22.5% higher than in WT, 19.5% higher than *phot1* and 26.3% higher than *uvr*_*8-2*_ (*P* ≤ 0.002, Fig. 2b). Moreover, the measured adaxial leaf index in *cry*_*1*_*cry*_*2*_*uvr*_*8-2*_ was on average 12.6% higher than in *cry*_*1*_*cry*_*2*_ (*P* = 0.014, Fig. 2b). In UV-B & No Blue treatment, the relative adaxial anthocyanin index in *uvr*_*8-2*_, *cry*_*1*_*cry*_*2*_ and *cry*_*1*_*cry*_*2*_*uvr*_*8-2*_ was on average 14.7% higher than in WT and *phot1* (*P* ≤ 0.007, Fig. 2c). In comparison, in UV-B & full PAR treatment, measurements in WT and *phot1* plants were similarly low (*P* = 0.438, Fig. 2d). The adaxial anthocyanin index measured in *cry*_*1*_*cry*_*2*_*uvr*_*8-2*_ was significantly higher than in any other genotype (*P* < 0.001), with the largest difference being 43.6% more than the WT (Fig. 2d). While the anthocyanin index in *cry*_*1*_*cry*_*2*_ was 18.2% lower than in *cry*_*1*_*cry*_*2*_*uvr*_*8-2*_ (*P* < 0001), it remained 22.8% higher than *uvr*_*8-2*_ (*P* < 0.001, Fig. 2d). The anthocyanin index was 10.6% higher in *uvr*_*8-2*_ than in WT and 6.1% higher than in *phot1* (*P* ≤ 0.046, Fig. 2d).

#### Relative leaf chlorophyll index

**Fig. 3:**
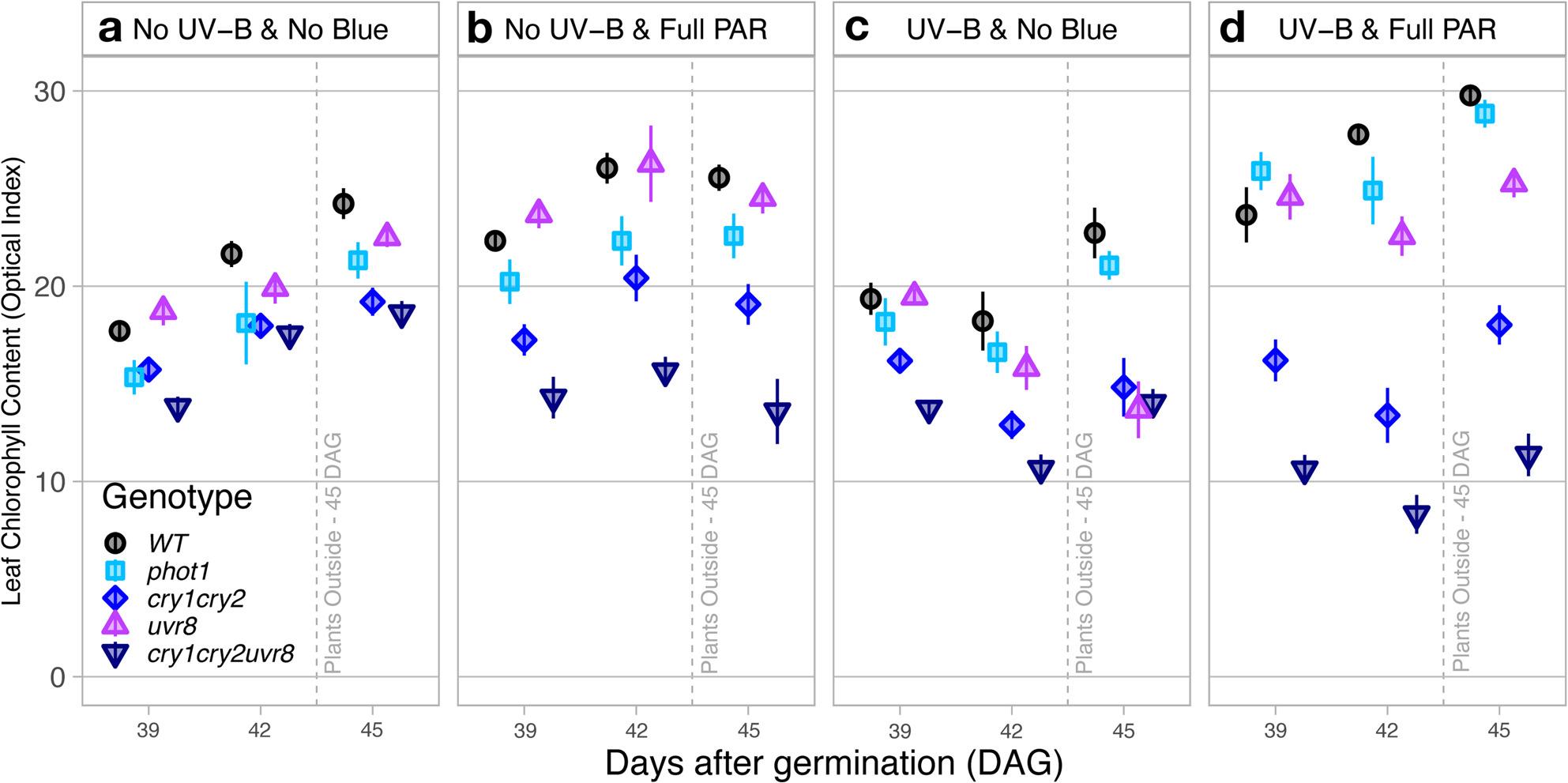
Chlorophyll content measured optically from the adaxial leaf side- Comparison of chlorophyll readings from the adaxial side obtained by Dualex in Arabidopsis wild-type and photoreceptor mutants growing under (**a**) No UV-B & No Blue, (**b**) No UV-B & Full PAR, (**c**) UV-B & No Blue and (**d**) UV-B & Full PAR and to natural sunlight exposure outdoors at midday. Plants were grown under Full PAR or No-Blue conditions from 11 to 35 days after germination, then split into two groups for an additional 5 h daily UV-B treatment over 9 days, with high-light stress applied on day 45. Values shown represent the mean chlorophyll index ± standard error (SE), calculated from plants grown in three replicate compartments for each treatment. Statistical analyses were performed using ANOVA; full tables are provided in Supplementary Tables S1 and S4

Leaf chlorophyll content significantly increased between measurements performed before and after sunlight exposure at 45 DAG for all genotypes (DAG|Genotype: *P* ≤ 0.007) except for *uvr*_*8-2*_ (*P* = 0.851). The trends found between genotypes in each treatment remained similar for all measurement days (Fig. 3a, b, c, d).

Similarly to adaxial flavonol and anthocyanin indices, treatment effects on the leaf chlorophyll index did not differ significantly between No UV-B & No Blue and No UV-B & full PAR for any genotype (*P* ≥ 0.058), except for *uvr*_*8-2*_ in which the chlorophyll index was 15.2% higher in No UV-B & full PAR than No UV-B & No Blue (*P* = 0.007, Fig. 3a & b). Interestingly, chlorophyll measurements in *cry*_*1*_*cry*_*2*_*uvr*_*8-2*_ were 19.5% lower in UV-B & No Blue compared to No UV-B & No Blue (*P* = 0.026) but no significant difference was found for other genotypes (*P* ≥ 0.060, Fig. 3a & c). In UV-B & full PAR conditions, the chlorophyll index was 30.4% higher on average compared to the same condition without blue light (UV-B & No Blue *P* ≤ 0.001) in WT, *phot1* and *uvr*_*8-2*_, but not in *cry*_*1*_*cry*_*2*_ and *cry*_*1*_*cry*_*2*_*uvr*_*8-2*_ (*P* ≥ 0.104, Fig. 3d).

Treatment effects on chlorophylls were also different depending on genotype. The Full PAR treatments, both with or without UV-B radiation (Fig. 3b & d), resulted in an increased difference (compared with the No Blue) in the relative leaf chlorophyll content among genotypes, in particular between WT and *cry*_*1*_*cry*_*2*_*uvr*_*8-2*_, where the chlorophyll index in WT was 61.6% higher than in *cry*_*1*_*cry*_*2*_*uvr*_*8-2*_ (*P* < 0.001). While the highest leaf chlorophyll indexes in No UV-B & Full PAR were in WT and *uvr*_*8-2*_ (*P* = 0.358), chlorophyll measurements were 8.8% lower in *phot1* (*P* ≤ 0.013), 20.7% lower in *cry*_*1*_*cry*_*2*_ (*P* ≤ 0.049) and 41.1% lower in *cry*_*1*_*cry*_*2*_*uvr*_*8-2*_ (*P* < 0. 001) compared to WT and *phot1* (Fig. 3b). In UV-B & No Blue conditions, although the leaf chlorophyll index was not significantly different between WT and *phot1* (*P* = 0.162), the chlorophyll index was 16.6% higher than in *uvr*_*8-2*_, 22.6% higher than in *cry*_*1*_*cry*_*2*_ and 30.9% higher than in *cry*_*1*_*cry*_*2*_*uvr*_*8-2*_ (*P* ≤ 0.023) (Fig. 3c). Exceptionally, the chlorophyll index decreased steadily over time in *uvr*_*8-2*_ when grown in UV-B without blue light (Fig. 3c). When exposed to both UV-B & full PAR, the chlorophyll index significantly differed between nearly all genotypes (*P* ≤ 0.001) with the exception of WT and *phot1* (*P* = 0.145), and *phot1* and *uvr*_*8-2*_ (*P* = 0.081, Fig. 3d). The highest average chlorophyll content was observed in WT, and was 2.9% higher than in *phot1*, 8.4% higher than in *uvr*_*8-2*_, 40.2% higher than in *cry*_*1*_*cry*_*2*_ and 61.6% higher than in *cry*_*1*_*cry*_*2*_*uvr*_*8-2*_ (Fig. 3d).

### Relationships between epidermal flavonols, anthocyanins, and chlorophyll contents in response to different light treatments

#### Relationships between chlorophyll and epidermal flavonol content

**Fig. 4:**
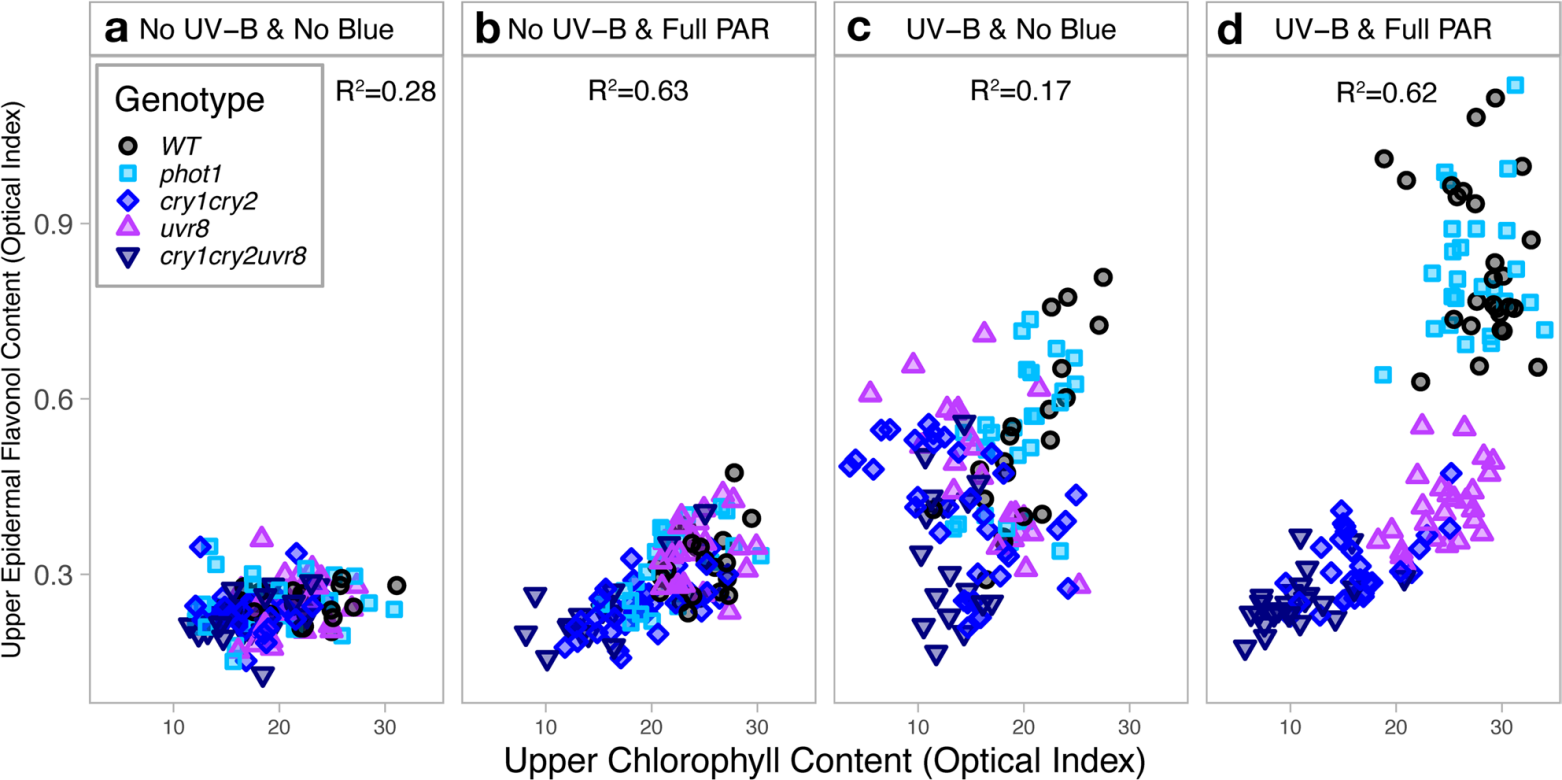
Relationships between leaf upper epidermal flavonols and leaf chlorophyll indexes across treatments - Dualex measurements in Arabidopsis wild-type and photoreceptor mutants growing under (**a**) No UV-B & No Blue, (**b**) No UV-B & Full PAR, (**c**) UV-B & No Blue and (**d**) UV-B & Full PAR. Each symbol represents the measurement for one individual plant. Statistical analyses were performed using Spearman rank correlation coefficient; full table is provided in Supplementary Table S5

Chlorophyll optical measurements were positively correlated with flavonol contents for all conditions (Fig. 4a, b, c, d). In No UV-B & full PAR (Fig. 4b) and UV-B & full PAR (Fig. 4d), there was a strong statistical correlation between chlorophyll and flavonol indexes (*R* = 0.63 and *R* = 0.62, *P* < 0.001). Lower flavonol index in *uvr*_*8-2*_, *cry*_*1*_*cry*_*2*_ and *cry*_*1*_*cry*_*2*_*uvr*_*8-2*_ were associated with lower chlorophyll index, while WT and *phot1* accumulated a higher chlorophyll index along with higher flavonol index (Fig. 4b & d). The positive correlation between chlorophyll and flavonol indexes were not as robust in No UV-B & No Blue (Fig. 4a) and UV-B & No Blue (Fig. 4c) than for plants grown full PAR treatments (Fig. 4b & d), where there was no particular trend associating chlorophyll and flavonol content between genotypes (*R* = 0.28 and *R* = 0.17, *P* ≤ 0.037).

#### Relationships between chlorophyll and epidermal anthocyanin content

**Fig. 5:**
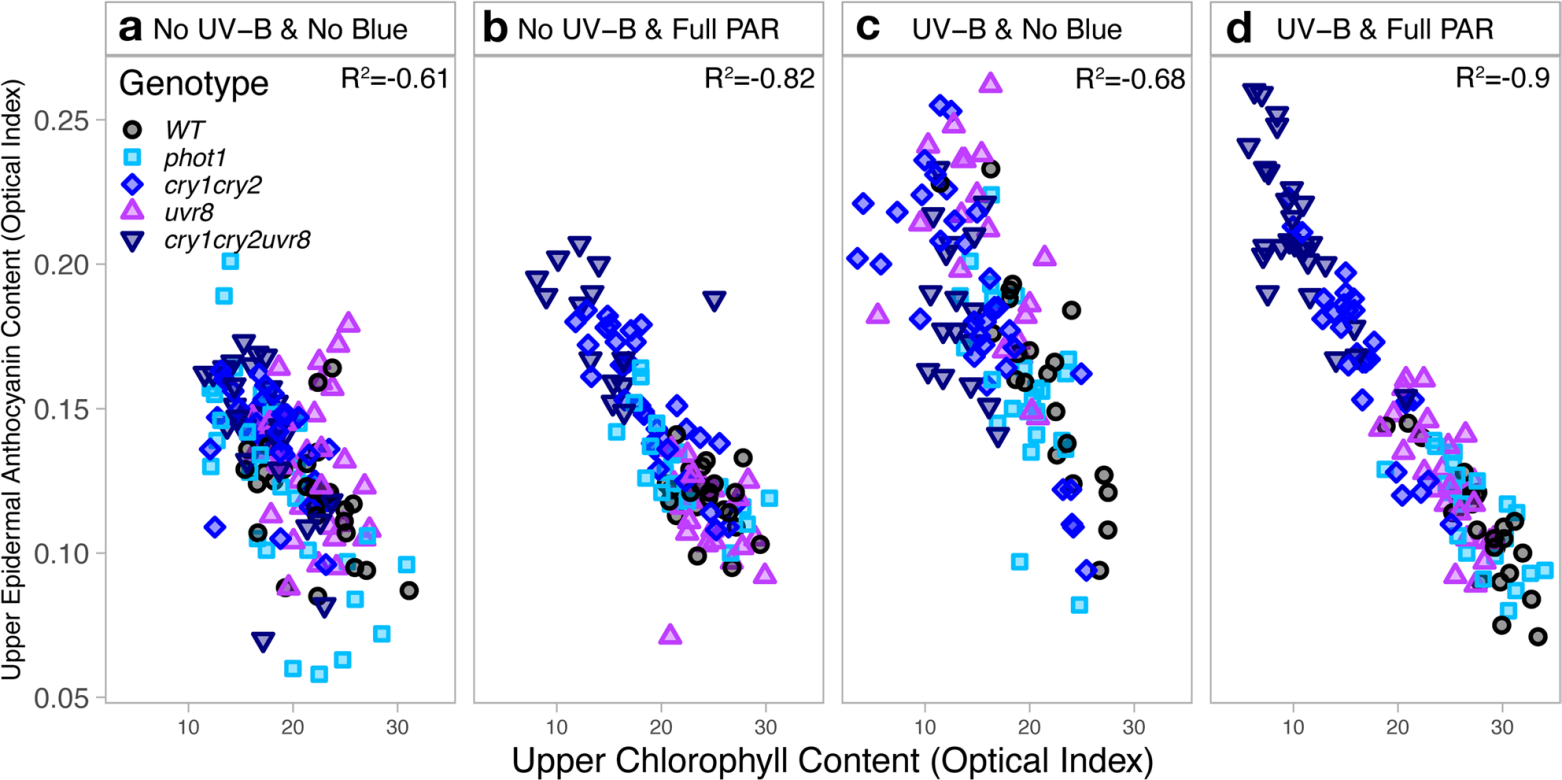
Relationships between leaf upper epidermal anthocyanin and leaf chlorophyll content - Dualex measurements in Arabidopsis wild-type and photoreceptor mutants growing under (**a**) No UV-B & No Blue, (**b**) No UV-B & Full PAR, (**c**) UV-B & No Blue and (**d**) UV-B & Full PAR. Each symbol represents the measurement for one individual plant. Statistical analyses were performed using Spearman rank correlation coefficient; full table is provided in Supplementary Table S5

A strong negative relationship was found between chlorophyll and anthocyanin optical measurements from the adaxial leaf across all light conditions (Fig. 5a, b, c, d). The correlation between chlorophyll and anthocyanin indexes was particularly strong among plants grown in UV-B & full PAR; wherein a lower epidermal anthocyanin index strongly correlated with a higher chlorophyll index (*R* = -0.9, *P* < 0.001, Fig. 5d). The negative correlation between chlorophyll and anthocyanin measurements was structured according to genotype, where the highest chlorophyll and lowest anthocyanin contents were in the WT and *phot1*, whilst the lowest chlorophyll but highest anthocyanin contents were in *cry*_*1*_*cry*_*2*_ and *cry*_*1*_*cry*_*2*_*uvr*_*8-2*_ (Fig. 5d). A similar trend and correlation was found in No UV-B & full PAR (*R* = -0.82, *P* < 0.001, Fig. 5b), as well as in No UV-B & No Blue (Fig. 5a) and UV-B & No Blue (Fig. 5c), although the correlation in absence of blue light was not as strong as that for plants grown in full PAR conditions (*R* = -0.61 and *R* = -0.68, *P* < 0 0.001).

#### Relationship between epidermal flavonols and anthocyanins

**Fig. 6:**
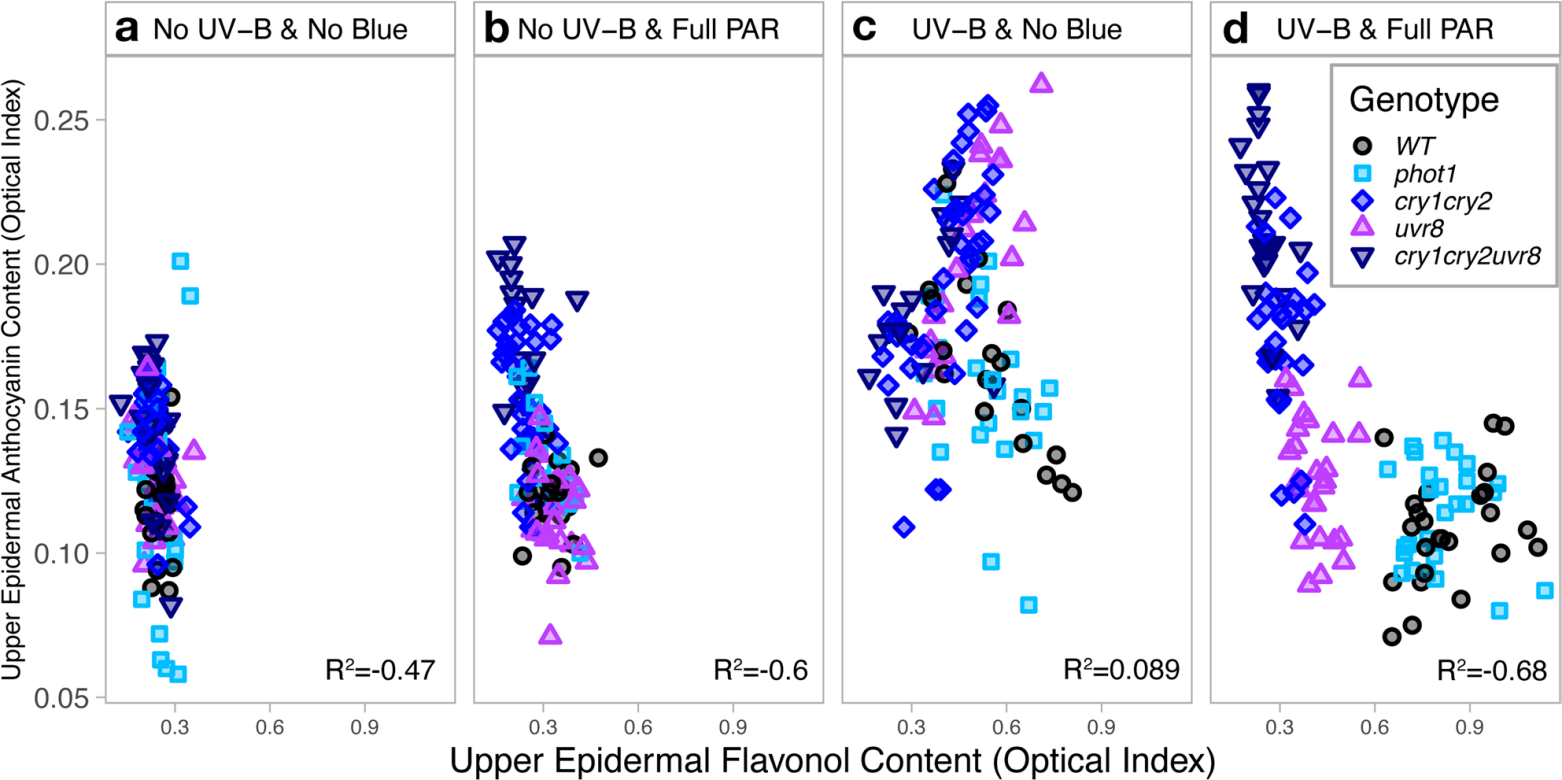
Relationships between leaf upper epidermal flavonols and anthocyanins indexes across treatments - Dualex measurements in Arabidopsis wild-type and photoreceptor mutants growing under (**a**) No UV-B & No Blue, (**b**) No UV-B & Full PAR, (**c**) UV-B & No Blue and (**d**) UV-B & Full PAR. Each symbol represents the measurement for one individual plant. Statistical analyses were performed using Spearman rank correlation coefficient; full table is provided in Supplementary Table S5

Epidermal flavonol and anthocyanin optical measurements were generally strongly correlated depending on light treatments (Fig. 6a, b, c, d). In UV-B & full PAR conditions, the negative correlation between flavonol and anthocyanin levels was particularly strong (*R* = -0.68, *P* < 0.001, Fig. 6d). While low anthocyanin contents were observed in genotypes that accumulated higher flavonol indexes, such as WT or *phot1;* the opposite trend was observed in *uvr*_*8-2*_, *cry*_*1*_*cry*_*2*_ and *cry*_*1*_*cry*_*2*_*uvr*_*8-2*_ genotypes where higher anthocyanin but lower flavonol indexes were measured (Fig. 6d). While statistically significant, the correlation between flavonol and anthocyanin indexes remained moderate under No UV-B & No Blue (Fig. 6a) and No UV- B & full PAR (Fig. 6b) conditions (*R* = -0.47 and *R* = -0.6, *P* < 0.001). Nevertheless, a grouping by genotype was apparent in both conditions with full PAR (Fig. 6b & d), but not in conditions without blue light, either with or without UV-B radiation (Fig. 6a & c). No significant correlation was present between flavonol and anthocyanin contents in UV-B & No Blue conditions (*R* = 0.089, *P* = 0.28, Fig. 6c).

### Effects of blue light and UV-B radiation during growth on chlorophyll fluorescence and the consequences for photochemical yield during exposure to bright sunlight

**Fig. 7:**
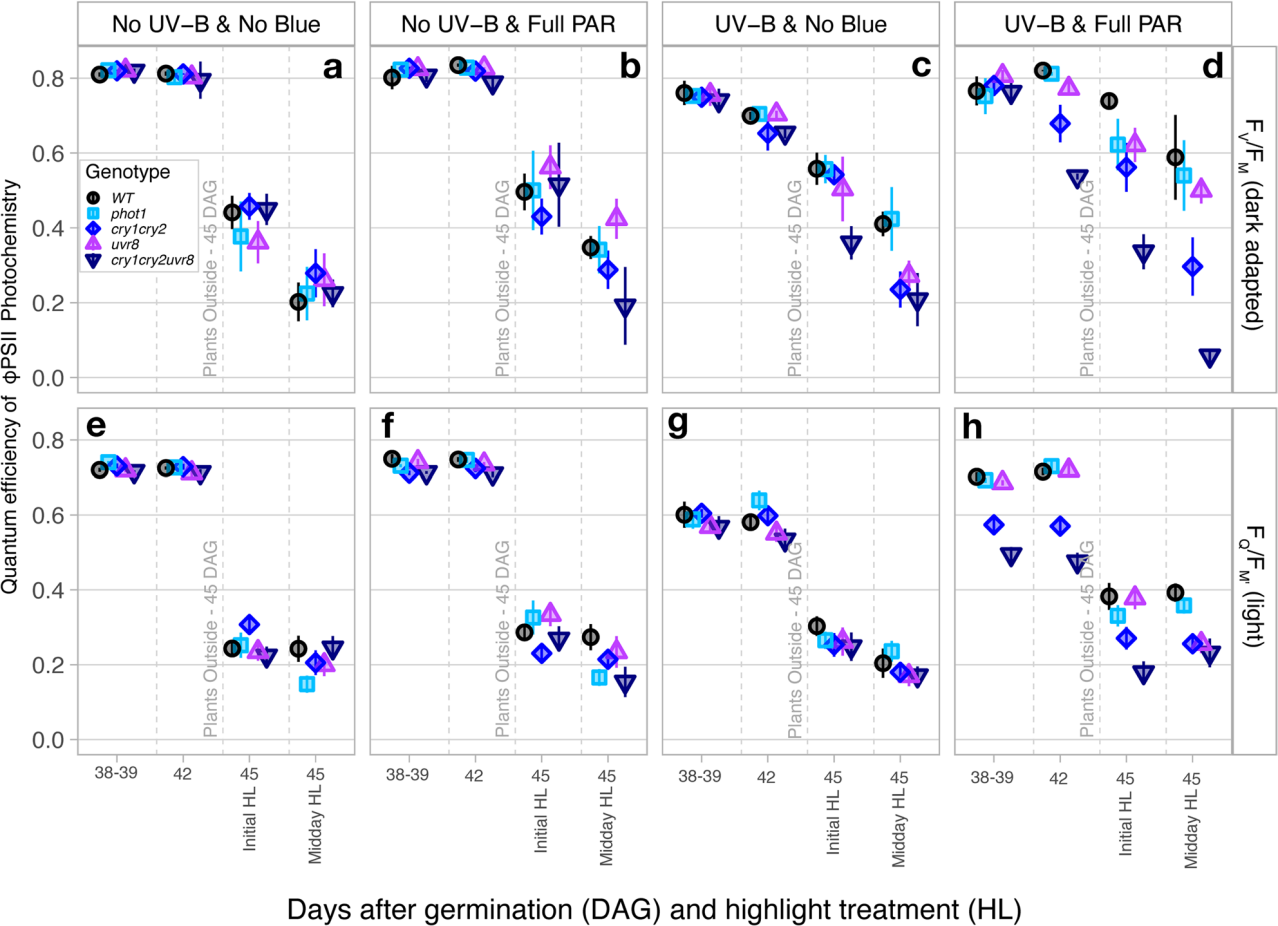
Comparison of the maximum quantum efficiency (F_V_/F_M_) and effective quantum yield of PSII (F_Q_/F_M_`) – (a – d) Maximum quantum efficiency of PSII was measured by chlorophyll fluorescence on Arabidopsis wild-type and photoreceptor mutants dark adapted leaves (see Methods), growing under (**a**) No UV-B & No Blue, (**b**) No UV-B & Full PAR, (**c**) UV-B & No Blue and (**d**) UV-B & Full PAR and to natural sunlight outdoors. Leaves were dark adapted for at least 30 min. (**e **– **h**) Effective quantum yield of PSII measured by chlorophyll fluorescence on Arabidopsis WT and photoreceptor mutants light adapted leaves (see Methods), growing under No UV-B & No Blue (**e**), No UV-B & Full PAR (**f**), UV-B & No Blue (**g**) and UV-B & Full PAR (**h**) and to natural sunlight outdoors. Values shown represent the mean chlorophyll index ± standard error (SE), calculated from plants grown in three replicate compartments for each treatment. Measurements were done on the same leaves as the Dualex measurements Figs. 1, 2 & 3. Statistical analyses were performed using ANOVA; full tables are provided in Supplementary Tables S1, S6 and S7

Overall, F_V_/F_M_ was significantly affected by the light treatment during growth depending on genotype (Treatment|Genotype: *P* < 0.001, Fig. 7a, b, c, d). Overall, high-light exposure had a negative impact on F_V_/F_M_ (*P* < 0.001), which differed according to genotype (*P* = 0.002). There was no significant combined effect of light treatment and time of the measurement (days after germination, initial highlight: 30 min after transfer to full sunlight; midday highlight: at solar noon 2–3 h after transfer, *P* > 0.095), nor three-way interaction (*P* > 0.118).

Only 42 DAG, before high light stress, did F_V_/F_M_ differ across different genotypes in UV-B & Full PAR treatment (Fig. 7d), when on average it was 30% lower in *cry*_*1*_*cry*_*2*_*uvr*_*8-2*_ (*P* < 0.001), than WT, while the other genotypes remained similar regardless of the light treatment (*P* ≥ 0.948). F_V_/F_M_ showed a modest decrease in *cry*_*1*_*cry*_*2*_ than in WT and *phot1* (*P* ≥ 0.050) and a modest increase compared to *cry*_*1*_*cry*_*2*_*uvr*_*8-2*_ (*P* = 0.050) but did not significantly differ from *uvr*_*8-2*_ (*P* = 0.211) prior high light stress (Fig. 7d).

The exposure to full sunlight depressed F_V_/F_M_ on average for all genotypes and light treatments, to 47.3% lower than prior sunlight exposure (Fig. 7a, b,c, d). However, F_V_/F_M_ during the initial response after 30 min of high-light stress did not significantly differ between genotypes when grown under conditions without UV-B radiation (*P* ≥ 0.550, Fig. 7a & b). In UV-B & No Blue treatment (Fig. 7c), F_V_/F_M_ was similar in WT, *phot1*,* uvr*_*8-2*_ and *cry*_*1*_*cry*_*2*_ (*P* ≥ 0.622), and on average 34.8% lower in *cry*_*1*_*cry*_*2*_*uvr*_*8-2*_ (*P* ≤ 0.012), but not statistically higher in *uvr*_*8-2*_ than in *cry*_*1*_*cry*_*2*_*uvr*_*8-2*_ *P* = 0.082). In UV-B & Full PAR (Fig. 7d), the initial response to high light stress, was similar in WT, *phot1* and *uvr*_*8-2*_ (*P* ≥ 0.175), and on average F_V_/F_M_ was 14.8% lower in *cry*_*1*_*cry*_*2*_ (*P* ≤ 0.005) and 49.1% lower in *cry*_*1*_*cry*_*2*_*uvr*_*8-2*_ (*P* < 0.001). Measured F_V_/F_M_ was also 40.7% higher in *cry*_*1*_*cry*_*2*_ than in *cry*_*1*_*cry*_*2*_*uvr*_*8-2*_ (*P* < 0.001).

At midday, no differences in F_V_/F_M_ between genotypes were recorded in plants grown under No UV-B & No Blue (Fig. 7a) and No UV-B & Full PAR (Fig. 7b) conditions (*P* ≥ 0.112), with the exception of a significantly higher F_V_/F_M_ in *uvr*_*8-2*_ than in *cry*_*1*_*cry*_*2*_*uvr*_*8-2*_ from the No UV-B & Full PAR treatment (*P* = 0.009). At midday, F_V_/F_M_ did not differ between WT and *phot1* (*P* = 0.847) plants from UV-B & No Blue (Fig. 7c), and was only marginally higher than in *uvr*_*8-2*_ (*P* ≥ 0.067), but was on average 47% higher than in *cry*_*1*_*cry*_*2*_ and 89.6% higher than in *cry*_*1*_*cry*_*2*_*uvr*_*8-2*_ (*P* ≤ 0.018). F_V_/F_M_ in *uvr*_*8-2*_, *cry*_*1*_*cry*_*2*_ and *cry*_*1*_*cry*_*2*_*uvr*_*8-2*_ grown in UV-B & No Blue (Fig. 7c) did not significantly differ at midday (*P* ≥ 0.481). In UV-B & Full PAR at midday (Fig. 7d), F_V_/F_M_ was similar in WT, *phot1* and *uvr*_*8-2*_ (*P* ≥ 0.175) and was on average 45.3% higher than in *cry*_*1*_*cry*_*2*_ (*P* ≤ 0.005) and 89.3% higher than in *cry*_*1*_*cry*_*2*_*uvr*_*8-2*_ (*P* < 0.001). F_V_/F_M_ in *cry*_*1*_*cry*_*2*_ was also 80.5% higher than in *cry*_*1*_*cry*_*2*_*uvr*_*8-2*_ (*P* < 0.001). Generally, the differences in F_V_/F_M_ between genotypes were accentuated by the high-light treatment, consistently the highest F_V_/F_M_ were measured in WT and *phot1*, followed by *uvr*_*8-2*_, *cry*_*1*_*cry*_*2*_ and finally *cry*_*1*_*cry*_*2*_*uvr*_*8-2*_ from all light treatments (Fig. 7a, b, c, d).

Light treatment prior high light stress had no significant influence on the F_V_/F_M_ regardless of genotype, both on the 38/39 DAG (*P* ≥ 0.775) and 42 DAG (*P* ≥ 0.082), with the exception of *cry*_*1*_*cry*_*2*_*uvr*_*8-2*_ for which F_V_/F_M_ was 27.9% lower in UV-B & Full PAR (Fig. 7d) on 42 DAG than in conditions without UV-B radiation (*P* ≤ 0.006, Fig. 7a & b). During the initial response to full sunlight exposure on 45 DAG, no statistically significant difference in F_V_/F_M_ was observed between light treatments for *cry*_*1*_*cry*_*2*_ and *cry*_*1*_*cry*_*2*_*uvr*_*8-2*_ (*P* ≥ 0.190, Fig. 7a, b, c, d). F_V_/F_M_ was 14.8% higher in *phot* and *uvr*_*8-2*_ treatment with UV-B and Full PAR (Fig. 7d) than in No UV-B & No Blue (Fig. 7a) conditions (*P* ≤ 0.022), but either full PAR or UV-B alone had no significant effect on F_V_/F_M_ (*P* ≥ 0.071).

During the initial high light stress response, F_V_/F_M_ in WT was on average 32.5% higher when grown in UV-B & Full PAR (Fig. 7d) than in any other treatment (*P* ≤ 0.034). At midday 45 DAG, still no difference in F_V_/F_M_ was observed between light treatments for *cry*_*1*_*cry*_*2*_ and *cry*_*1*_*cry*_*2*_*uvr*_*8-2*_ (*P* ≥ 0.132). F_V_/F_M_ in *uvr*_*8-2*_ was 45.6% higher in UV-B & Full PAR than in UV-B & No Blue (Fig. 7c) and 47.8% higher than in No UV-B & No Blue (*P* ≤ 0.019, Fig. 7a) but did not significantly differ within the other light treatments (*P* ≥ 0.082). Treatment with UV-B & Full PAR (Fig. 7d) produced a 47.6% higher F_V_/F_M_ at midday in *phot1* compared to treatments without UV-B (*P* ≤ 0.032) but did not differ between other light treatments (*P* ≥ 0.074). F_V_/F_M_ in WT was on average 53.3% higher when grown in conditions with UV-B (Fig. 7c & d) than in conditions without UV-B (*P* < 0.001, Fig. 7a & b), but the addition of Full PAR alone had no significant effect on F_V_/F_M_ (*P* ≥ 0.122, Fig. 7b).

Considering the effective quantum yield of PSII in ambient light (F_Q_/F_M’_), it was significantly impacted by the interaction of light treatment and genotype, as well as by the light treatment - timing of measurement interaction (*P* = 0.001). No significant combined effect of genotype and time of the measurement, nor three-way interaction was recorded (*P* ≥ 0.135), but light treatment alone had a small effect (*P* = 0.029) (Fig. 7e, f, g, h).

Before high-light stress, there was no significant differences in F_Q_/F_M’_ between genotypes in any light treatment, with the only exception of plants acclimated to growth in UV-B & Full PAR (*P* ≥ 0.232, Fig. 7h) where WT, *phot1* and *uvr*_*8-2*_ were on average 28.6% higher than in *cry*_*1*_*cry*_*2*_*uvr*_*8-2*_ on the 38/39 DAG and by 34.1% on 42 DAG (*P* ≤ 0.008). By 42 DAG, F_Q_/F_M’_ in *cry*_*1*_*cry*_*2*_ plants under UV-B & Full PAR (Fig. 7h) treatment was also significantly (20.9%) lower than in WT, *phot1* and *uvr*_*8-2*_, but higher (16.7%) than in *cry*_*1*_*cry*_*2*_*uvr*_*8-2*_ (*P* ≤ 0.037).

During the initial response to high-light stress outside in full sunlight 45 DAG, F_Q_/F_M’_ was overall not significantly different between genotypes from the No UV-B & No Blue (Fig. 7e), nor No UV-B & Full PAR (Fig. 7f), treatments (*P* ≥ 0.117), with the exception of a slightly higher F_Q_/F_M’_ in *cry*_*1*_*cry*_*2*_ compared to *cry*_*1*_*cry*_*2*_*uvr*_*8-2*_ from No UV-B & No Blue (*P* = 0.041, Fig. 7e), and a lower F_Q_/F_M’_ in *cry*_*1*_*cry*_*2*_ compared to *phot1* and *uvr*_*8-2*_ from No UV-B & Full PAR (*P* ≤ 0.043, Fig. 7g). In plants from UV-B & Full PAR (Fig. 7h), F_Q_/F_M’_ was 36.5% higher in WT, *phot1* and *uvr*_*8-2*_ than in *cry*_*1*_*cry*_*2*_*uvr*_*8-2*_ (*P* < 0.001) during the initial high-light. Although, F_Q_/F_M’_ was 29% lower in *cry*_*1*_*cry*_*2*_ than in WT and 28.3% lower than in *uvr*_*8-2*_ (*P* ≤ 0.009), it did not differ significantly from *phot1* (*P* = 0.175), but *cry*_*1*_*cry*_*2*_ was 39.4% higher than in *cry*_*1*_*cry*_*2*_*uvr*_*8-2*_ (*P* = 0.010).

At midday, differences in F_Q_/F_M’_ between genotypes were only detected in plants from UV-B & Full PAR treatment (*P* ≥ 0.056, Fig. 7h), except for a lower F_Q_/F_M’_ in *phot1* compared to WT from the No UV-B & Full PAR (*P* = 0.044, Fig. 7f). At midday, plants from UV-B & Full PAR (Fig. 7h), had a similar F_Q_/F_M’_ between WT and *phot1* (*P* ≥ 0.443), but on average this was 34% higher than in *uvr*_*8-2*_, *cry*_*1*_*cry*_*2*_ and *cry*_*1*_*cry*_*2*_*uvr*_*8-2*_ (*P* ≤ 0.012). F_Q_/F_M’_ was not significantly different in *uvr*_*8-2*_, *cry*_*1*_*cry*_*2*_ and *cry*_*1*_*cry*_*2*_*uvr*_*8-2*_ at midday during high light stress (*P* ≥ 0.458).

F_Q_/F_M’_ was not affected by any light treatment in *phot1* at any time prior to high-light stress (*P* ≥ 0.156). Under UV-B & No Blue (Fig. 7g), F_Q_/F_M’_ in the WT and *uvr*_*8-2*_ plants was respectively 21.1% and 23.8% lower than in the other light treatments, although the magnitude of the effect was moderate (42 DAG, *P* ≤ 0.036; 38 DAG *P* ≥ 0.075). The combination of UV-B & Full PAR produced similar F_Q_/F_M’_ to conditions without UV-B radiation in WT and *uvr*_*8-2*_ plants (*P* ≥ 0.531, Fig. 7h). On 38/39 DAG, F_Q_/F_M’_ in UV-B & Full PAR-grown plants (Fig. 7h), compared to treatments without UV-B (Fig. 7e & f), was 30.8% lower in *cry*_*1*_*cry*_*2*_*uvr*_*8-2*_ (*P* ≤ 0.027) but only marginally lower in *cry*_*1*_*cry*_*2*_ (*P* ≥ 0.045). However, on the 42 DAG, F_Q_/F_M’_ was 19.6% lower in *cry*_*1*_*cry*_*2*_ and 29.8% lower in *cry*_*1*_*cry*_*2*_*uvr*_*8-2*_ in both treatment with UV-B radiation (Fig. 7g & h) compared to those without (Fig. 7e & f) (*P* ≤ 0.006).

When exposed to full sunlight, F_Q_/F_M’_ was not significantly different between light treatments in *cry*_*1*_*cry*_*2*_ and *cry*_*1*_*cry*_*2*_*uvr*_*8-2*_, neither during the initial response nor at midday on 45 DAG (*P* ≥ 0.216). F_Q_/F_M’_ was 34.4% higher in *uvr*_*8-2*_ from UV-B & Full PAR than growing conditions without blue light, during the initial high light response (*P* ≤ 0.034, Fig. 7h), but remained similarly suppressed at midday regardless of prior light treatment (*P* ≥ 0.351). F_Q_/F_M’_ in WT and *phot1* were similar to each other irrespective of prior light treatment during the initial high-light stress response (*P* ≥ 0.080), with the exception of a 36.4% higher F_Q_/F_M’_ in plants from UV-B & Full PAR (Fig. 7h) than from No UV-B & No Blue (Fig. 7e) for WT (*P* = 0.006). At midday, F_Q_/F_M’_ in WT and *phot1* acclimated to UV-B & Full PAR had a respectively 38.9% and 48.9% higher effective quantum yield than in any other light condition (*P* ≤ 0.014).

## Discussion

### How did transfer outdoors into full sunlight affect photosynthesis?

To test the role of photoreceptors in conferring resistance to acute high-light stress, we used mutants *cry*_*1*_*cry*_*2*_, *uvr*_*8-2*_, *phot1* and triple *cry*_*1*_*cry*_*2*_*uvr*_*8-2*_*vs* the WT, and assessed these plants’ photosynthetic capabilities on transfer to sunlight. We hypothesized that the response mediated by cryptochromes and UVR8 photoreceptors contributes to better maintenance of photosynthetic capacity when plants are exposed to sudden increase in irradiance on transfer outside to full sunlight. Following transfer to full sunlight, optically estimated chlorophyll content increased in most genotypes. These rapid changes in leaf optical properties may reflect pigment organization rather than an increase in chlorophyll pools [34]. Consistent with this interpretation, F_M’_ decreased strongly after transfer to full sunlight (Figure S1), particularly at midday, indicating increased excitation pressure rather than enhanced PSII efficiency [35]. We found that the response mediated by cryptochromes allowed for a higher photosynthetic yield upon transfer to full sunlight, compared with UVR8 or PHOT1. Cryptochromes have been reported to regulate the turnover and repair of D1 and D2 proteins, which are involved in the PSII repair cycle [36], consequently facilitating the replacement of photodamaged PSII reaction centers, thereby limiting photoinhibition and maintaining high photosynthetic yield. More generally, cryptochromes contribute to developmental processes, especially responses to fluctuating light conditions [37]. They activate gene expression in response to excessive PAR irradiance, UV-B and UV-A radiation independently of UVR8 [22, 31, 38]. This broad spectrum of actions mediated by cryptochromes confers tolerance to high-light stress in terms of photosynthetic capacity.

Surprisingly, the photosynthetic yield of UVR8 mutants equaled that of WT plants, however the loss of both UVR8 and cryptochromes led to its severe decline (Fig. 7). Thus, while there is some redundancy allowing the plant to accommodate deficiencies in either CRY or UVR8, these two photoreceptors together likely play a central role in maintaining PSII integrity by limiting PSII damage upon exposure to high irradiance. At the molecular level, cryptochromes and UVR8 are both involved in the modulation of the transcription factor HY5 [21], known to upregulate the precursor gene of the flavonol biosynthetic pathway *CHALCONE SYNTHASE (CHS)* [39, 40]. This prevents excess cellular damage associated with UV and light stress [22, 41]. The close interaction of UVR8 and cryptochromes in plants grown in sunlight and field conditions is in agreement with recent findings [16, 21, 42], moreover cryptochromes are able to modulate UVR8 expression [21]. However, here we reveal that they are also integral to the response following a transition from low to high light. Their combined action allows a fine-tuned response to high-light stress, maintaining photosynthetic capacity and preventing PSII damage [21].

Interestingly, our results provided no evidence that PHOT1 contributed to the maintenance of photosynthetic yield through a cryptochrome-mediated pathway, which would have altered photosynthetic responses in *phot1* under conditions where CRY signaling is active, nor did PHOT1 prevent photoinhibition in response to high-light stress. This suggests that PHOT1 acts independently of cryptochrome signaling in the regulation of photosynthetic performance under high-light conditions. PHOT1 has been found to be negatively regulated by cryptochromes in response to blue light, promoting the accumulation of anthocyanins and decreasing responsiveness to saturating light [43]. Thus, the mutation of PHOT1 might have removed the need for cry to downregulate transcripts in the first place. Importantly, this transcriptional regulation does not preclude a physiological role for PHOT1 under blue light, as PHOT1 also controls chloroplast repositioning in response to blue irradiance [44]. Through blue-light–induced chloroplast accumulation, PHOT1 can modulate light absorption, which could influence susceptibility to photoinhibition. Chloroplast accumulation is generally associated with enhanced light capture under low irradiance, whereas chloroplast avoidance responses under high light are linked to photoprotection. Unlike PHOT1, the phototropin PHOT2 has been reported to predominantly act in avoidance of high-light stress, by promoting chloroplast movement [45, 46]. PHOT2 might play a bigger role in the maintenance PSII efficiency and preventing PSII damage upon high light stress than PHOT1. On the other hand, PHOT1 and PHOT2 are highly redundant [47, 48], which suggests that at well as being more involved in responses to high light stresses, the action of PHOT2 may have compensated for the loss of PHOT1 in our experiment.

### How did prior growth under blue light and UV-B radiation affect adjustment to sunlight outdoors?

We hypothesized that growing *Arabidopsis thaliana* plants under PAR treatments including blue light and UV-B radiation together would prime these plants for transfer to sunlight. The presence of these short wavelength regions during growth may allow for faster activation those processes that when upregulated improve acclimation to high light, by reducing the loss of photosynthetic yield and PSII damage [49]. Our results suggest that this was the case, as growth under PAR including blue light allowed for reduced photosynthetic suppression and maintained PSII operating efficiency of plants after exposure to sunlight compared to plants grown without blue light. The removal of blue light in the no-blue treatment also reduced total PAR relative to the control. Such changes in growth irradiance have been shown to affect plant growth and photosynthetic performance [50, 51]. Consequently, responses under no-blue conditions may reflect both changes in light quality and quantity. Blue light is generally considered as a crucial component of PAR for efficient photosynthesis, and numerous studies have found it to increase photosynthetic capacity [52–57]. The importance of blue-light for acclimation to high light stress has been described in several species, including *Arabidopsis thaliana*,* Spinacia oleracea* (spinach), and the algae *Phaeodactylum tricornutum* [58–60]. Likewise, blue light confers better recovery from exposure to UV-B radiation in *Capsicum annuum* (pepper) plants [61], and, while pre-treatment with full PAR alone did not prevent a decline in F_V_/F_M_ and φPSII after high-light exposure as efficiently as growing conditions with addition of UV-B radiation, exposure to either full PAR or natural sunlight during growth does contribute to better light stress tolerance [22, 31, 50]. Part of the variation in chlorophyll content may also reflect growth-related changes during plant development, particularly in mutants with reduced growth rates. However, the response of chlorophyll content to light treatment suggests that this effect is less influential than the imposed UV and light environments.

On the other hand, conditioning by growth in UV-B radiation in absence of blue light during growth did reduce F_V_/F_M_ prior the high-light stress. This likely indicates either non-reversible damage to the photosystem, slower damage repair or damage to the repair machinery itself. Without the activation of blue-light photoprotection responses during growth, the prolonged UV-B treatment plants received is likely to have constituted a stressor. This would explain the depression of PSII yield during growth in these plants. The decrease in F_V_/F_M_ and φPSII caused by transfer of these plants from growth conditions to full sunlight was nevertheless smaller than that of plants not grown under UV-B radiation. This decrease in photosynthetic yield was also smaller in plants grown the PAR treatment including blue light, which is known to stimulate UV-B stress recovery [62]. Unlike high doses of UV-B, which can induce stress symptoms, using moderate doses of UV-B radiation can promote the expression of stress response genes and the accumulation of ROS scavenging compounds, hence increasing stress tolerance [24, 63, 64]. This may have allowed allowed for the accelerated response to the acute stress caused by transfer to sunlight that we report.

The presence of both full PAR and UV-B radiation in the spectral treatments during growth produced values of F_V_/F_M_ on exposure to sunlight higher to those found in plants grown without UV-B. Thus, this spectral combination also partially mitigated the decline in F_V_/F_M_ and φPSII on transfer to sunlight despite the light stress incurred. The combination of both UV-B radiation and full PAR did provide the best conditions for plants to adjust their photosynthesis under sunlight. PAR, in particular blue light, and UV-B radiation have been found to trigger complimentary response pathways [7]. Growth under both spectral regions activates a wider range of photoreceptors, in this case cryptochromes and UVR8 [7], which mediate stress responses [7], essential to maintain F_V_/F_M_,φPSII, conferring protection against high-light stress [65]. It should be noted that recent mechanistic work has shown that F_V_/F_M_ does not represent the true maximum quantum yield of PSII photochemistry, as true Fm is achieved through the formation of a light-adapted charge-separated state (PSII_L_) rather than simple reaction-center closure [66]. Consequently, differences in F_V_/F_M_ between mutants or light treatments may reflect underlying structural and functional dynamics of PSII, which warrant further investigation.

In *Arabidopsis thaliana*, additional UV-B treatment during growth has been found to limit decline in photosynthetic yield, and to enable higher carbon assimilation rate under saturating light conditions, while enhancing cross-tolerance to a range of abiotic stresses, such as drought and heat [23, 67, 68]. This priming effect of UV radiation has been widely applied in agriculture and horticulture to enhance resistance to biotic and abiotic stresses commonly impacting crop production [65, 69]. Here, we found that growth under broad spectrum of radiation enhances the capacity of plants to adjust to a new environment, for instance to fluctuating light conditions [22, 61].

### Was that improved capacity for adjustment conferred under pre-treatments with blue light or UV-B radiation unavailable to certain knock-out mutants?

The priming response through full PAR and UV-B radiation during growth was largely unavailable to *cry*_*1*_*cry*_*2*_ and the triple mutant *cry*_*1*_*cry*_*2*_*uvr*_*8-2*_, while *uvr*_*8-2*_ mutant remained able to benefit to some extent from the blue light provided in full PAR, to prime in anticipation of high-light stress. Hence, we suggest that the priming for high light conferred through the presence of the full spectrum of solar radiation at low irradiances during growth is largely underwritten by the response mediated through cryptochromes and their ability to activate stress response pathways. On the other hand, priming for high light was still available to *uvr8* mutants to a greater extent than cryptochrome and triple mutants, it was evident that *uvr8* plants could not benefit from the extra protection conferred by the UVR8 response under UV-B radiation pre-treatment. This can be attributed to the respective action spectrum of cryptochromes and UVR8: while UVR8 mostly absorb in wavelengths below 315 nm [70], cryptochromes have shown to absorb wavelengths down to 300 nm [7]. Our UV-B treatment did emit in the UV-A region as well, hence, a stress response to both UV-A and full PAR mediated by cryptochromes might have taken place in *uvr8* mutants. Moreover, the light response mediated by cryptochromes affects a broader range of developmental processes besides stress responses: the regulation of seedling de-etiolation [71], the circadian clock [72, 73], stomatal openings [74, 75] as well as the regulation of other photoreceptors such as phytochromes or UVR8 [7, 73].

The enhanced anthocyanin accumulation observed mainly in the *cry*_*1*_*cry*_*2*_ and *cry*_*1*_*cry*_*2*_*uvr*_*8-2*_ mutants may represent a compensatory stress-response strategy that prioritizes photoprotection, potentially at the expense of growth and photosynthetic investment. Indeed, anthocyanin biosynthesis is tightly connected to carbon status: for example, sucrose availability is a strong inducer of anthocyanin accumulation via transcriptional control [76]. At the same time, photoreceptors are known to sit at major regulatory hubs (COP1/SPA, PIF networks) that coordinate growth and defence allocation decisions, making such trade-offs a possible outcome of altered photoreceptor signalling [1].

### Was there a relationship between flavonol content and PSII efficiency?

Priming through growth under spectra including blue light and UV-B radiation resulted in a greater upper epidermal flavonol content than in their absence. We found that those plants primed and accumulating more epidermal flavonols were associated with both higher F_V_/F_M_ and φPSII before high light stress and reduced inhibition of PSII efficiency during high-light stress. Mutants deficient in cryptochrome, UVR8 and both these photoreceptors, behaved similarly, whereby lower flavonol content corresponded to overall loss of PSII efficiency during and after high-light stress. The accumulation of photoprotective compounds has been found to largely rely on the response to both blue light and UV-B radiation through cryptochromes and UVR8 photoreceptors, consistent with Morales et al. (2025) [16], who found CRY–UVR8 co-activation maintains PSII yield under UV-blue exposure. These photoreceptors have repeatedly been found to increase the expression of flavonol biosynthesis genes, such as the flavonol precursor *CHALCONE SYNTHASE*, which promotes flavonol accumulation in response to blue light, UV-A and UV-B radiation [7, 39, 77–79]. Flavonols act as photoprotective compounds by absorbing UV radiation, attenuating UV penetration into leaf tissues and thereby indirectly limiting UV-induced oxidative stress, and hence preventing cellular damage [80–82]. Although flavonol accumulation occurs dynamically throughout the day in natural conditions, the associated molecular response is initiated within minutes-to-hours and potentially continues beyond the initial stress [7, 20]. Our study concurs with other reports finding that the accumulation of epidermal flavonols in response to UV-B leads to increased photoprotection and maintenance of photosynthetic yield during high light stress [83, 84].

Interestingly, while the accumulation of flavonols was closely linked to the photoreceptor responses to blue light and UV-B radiation, epidermal anthocyanins were accumulated when this response was weakened, in an inverse correlation to flavonol accumulation. Both flavonol and anthocyanin biosynthesis originate from the same biosynthetic pathway and hence share the same pool of precursors [85]. In plants where flavonol accumulation takes place as response to priming or stress, the pool of precursors available for anthocyanin biosynthesis might be reduced, and could limit anthocyanin accumulation in the short term. When both mechanisms are available, flavonols may accumulate in priority as screening compounds to prevent light stress damage [82, 86], even though anthocyanins could provide better antioxidant and ROS scavenging functions in response to high-light stress [87–89]. In absence of screening compounds, UV radiation and high-light stress can be a source of oxidative stress. Besides potentially acting as ROS scavenging compounds, anthocyanins may be synthesized in response to ROS accumulation upon stress [90], independently of light and photoreceptors responses [91]. Enhanced anthocyanin accumulation in absence of flavonols could therefore be a consequence of an accumulation of ROS when photoprotection is not ensured. Some studies have demonstrated shielding capacities of anthocyanins in red leaves in particular during autumn senescence [92, 93]. In addition, anthocyanins can absorb a proportion of incident blue and green light, thereby reducing the photon flux reaching chloroplasts, which may be relevant under our light conditions [94].

## Conclusions

This study reveals that coordinated signalling by blue- and UV-sensitive photoreceptors, particularly cryptochromes and UVR8, underlies the capacity of *Arabidopsis thaliana* to endure abrupt transitions from low to high irradiance. Pre-exposure to these shortwave regions primes photoprotective mechanisms, most notably the accumulation of epidermal flavonols and the maintenance of PSII efficiency, enabling a rapid, integrated response to sunlight stress. Our findings demonstrate that cryptochromes serve as pivotal regulators in this process, while their interplay with UVR8 fine-tunes protection against photoinhibition. Together, these photoreceptors orchestrate a spectrum-wide strategy linking developmental light sensing with acute stress resilience. Our study thus highlights the importance of blue- and UV-B–mediated photoreceptor signalling in priming photoprotection under a non-constant light environment (Table 1), such as those commonly experienced by field-grown crops. Strategies that preserve or enhance CRY- and UVR8-dependent signalling through breeding, spectral management, or canopy light optimization, may therefore improve the capacity to maintain PSII efficiency and reduce photoinhibition under dynamic light regimes.

**Table 1:** Conceptual model summarizing genotype-specific responses to blue and UV priming, showing relative changes in flavonols, anthocyanins, and photoinhibition compared to no priming conditions. Blue light priming reduces photoinhibition through CRY-dependent signalling, while UV-B provides an additive benefit only when blue signalling is intact; in the absence of CRY, responses shift toward anthocyanin accumulation without reduction of photoinhibition. Arrows up and down reflect an increase and a decrease, respectively. No arrow means no change relative to responses without priming

Genotype	Priming	Flavonols	Anthocyanins	Photoinhibition
WT & *phot*_*1*_	No priming			
	Blue priming	↑		↓
	UV priming	↑↑	↑	↓
	UV & Blue priming	↑↑↑		↓↓
*uvr*_*8−2*_	No priming			
	Blue priming	↑		↓
	UV priming	↑	↑	
	UV & Blue priming	↑		↓
*cry*_*1*_ *cry*_*2*_	No priming			
	Blue priming			
	UV priming		↑	
	UV & Blue priming		↑	
*cry*_*1*_ *cry*_*2*_ *uvr*_*8−2*_	No priming			
	Blue priming			
	UV priming		↑	
	UV & Blue priming		↑↑	↑↑

## Materials and methods

### Plant material and growth conditions

The priming response of growth under different light treatments was tested in WT accession Landsberg *erecta* (L*er*) of *Arabidopsis thaliana*, the previously characterized photoreceptor mutants *phot1* [95], *uvr*_*8-2*_ [96] and the double mutant *cry*_*1*_*cry*_*2*_ [97]. Genotyping of the triple mutant *cry*_*1*_*cry*_*2*_*uvr*_*8-2*_ was done as described in Rai et al., 2019 [22]. Seeds were placed in 0.1% agarose to imbibe moisture for 24 h at room temperature before being placed at 4 °C for 3 days for stratification to improve synchrony of germination and germination rate. The seeds germinated in 6-x-6-cm pots in peat and vermiculite (1:1) in a growth room (19 °C at night, 23 °C the day; 12 h/12 h day cycle, at a constant light intensity at 250 µmol m^-2^ s^-1^, 70% relative humidity) and at 11 days-old individual seedlings were transplanted to 8-x-8-cm pots containing the same composition of substrate. The experimental setup was designed with six identical compartments in a temperature-controlled growth room: four plants of each genotype were placed in each compartment.

The experiment was preceded by two pilot experiments performed in the same growth room, to make initial assessments of treatments effects over time and according to the dose of UV-B radiation and PAR. These experiments allowed us to refine the experimental design to give appropriate irradiance treatments to induce moderate changes in photoprotection.

### Irradiance treatments

During growth, plants received two different irradiance treatments from broad-spectrum LED lamps (Valoya AP67, 400–750 nm, PAR 169 µmol m^-2^ s^-1^ plus 32 µmol m^-2^ s^-1^ far red). Three compartments received the full lamp spectrum including 24 µmol m^-2^ s^-1^ blue light (henceforth “Full PAR”), while the three other compartments received the same light spectrum except with blue light attenuated (henceforth “No Blue”), created using a blue-light-attenuating film covering the LED lamps (Rosco 313 Canary Yellow Supergel; Westlighting, Helsinki, Finland). The number and height of lamps were adjusted so that plants in all compartments received similar total energy irradiance and the compartments were well ventilated to maintain constant temperature. The day period lasted for 10 h, the night period 14 h. The daytime temperature was set to 15 °C for the first 3 weeks, and then to 20 °C for the remainder of the experiment, while the night-time temperature was about 4 °C lower than the day throughout the experiment.

After 35 days after germination, when plants of all genotypes had at least four fully expanded leaves allowing measurements to be made, plants from each compartment were divided randomly into two groups. The growth room was reconfigured to create 12 compartments, half of which received broadband UV-B radiation (UV-B-313-nm EL, Q-Lab Europe, Ltd., Bolton, England). These compartments were separated from the other half that received no UV-B radiation by using UV-B-attenuating polyester curtains. The UV-B treatment lasted for nine days in combination with Full PAR or No Blue treatments in a fully factorial design. The UV-B radiation treatment gave a biologically effective energy irradiance of 0.196 W m^-2^ s^-1^ (equivalent to 0.491 µmol m^-2^ s^-1^ photon irradiance), similar to full sunlight around midday on a sunny summer’s day in Helsinki, and lasted for 5 h daily, giving a total daily dose of 3.52 kJ m^-2^ day^-1^ (see [98]) weighted according to Green’s formulation of the generalised plant action spectrum (GEN G; [99]). The purpose of this UV-B radiation treatment was to stimulate the accumulation of UV-screening flavonols in the WT plants of *A. thaliana* without causing them substantial damage, rather than to simulate natural conditions, since it constituted a higher dose than would be expected in nature relative to the amount of PAR received by the plants under the LED treatments. Published studies of flavonol accumulation in *Arabidopsis thaliana* indicate that this irradiance of UV-B radiation would be appropriate for this purpose (e.g. Morales et al., 2013 [100]).

### Optical measurements of leaf pigments and chlorophyll fluorescence

Leaf chlorophyll and the epidermal anthocyanin and flavonol contents were measured non-invasively with the Dualex Scientific^+^ (Force A, Paris, France). The upper side of two fully expanded leaves per plant were measured, to obtain values based on an optical index of epidermal flavonols and anthocyanins in the adaxial epidermis [101].

Chlorophyll fluorescence was measured using a mini-PAM (Heinz-Walz, Berlin, Germany). This involved, measurements of the effective quantum yield (φPSII or F_Q_/F_M_`) in the growing conditions with the LED lamps on, paired with measurements of the maximum quantum yield of photosystem II (F_V_/F_M_) of those same leaves measured in the same location. Recordings of F_V_/F_M_ were made after at least 30 min dark adaptation of photosynthesis using darkening clips, DLC-8 (Heinz-Walz, Berlin, Germany). Optical trait measurements were all done 38/39 DAG and 42 DAG, after plants spent one week under the UV-B treatments.

### Acclimation to full sunlight exposure outdoors

To test the priming effect of growth with blue light and/or UV-B radiation for acclimation to full sunlight, all plants were transferred outdoors into full sunshine on the 4th of May 2017 at 10:30 a.m. on a cloudless day at the end of the experiment (at 45 DAG). The effects of the increase in irradiance on chlorophyll fluorescence were measured within 30 min of transfer to full sun (initial measurement), and around solar noon (13:18 local time, midday measurement) approximately 120–150 min after the plants were transferred outdoors. Paired measurements of φPSII and F_V_/F_M_ were made as described above. Following these measurements, a Dualex reading from the upper side of the leaves was taken after 4 h had elapsed outdoors in full sun. The spectral energy irradiance at solar noon was 350 W m^-2^ PAR (i.e., photosynthetic photon flux density of 1600 µmol m^-2^ s^-1^) on this cloudless day (4th May 2017); the biologically effective dose of UV-B energy irradiance was 0.127 W m^-2^ (GEN G weighted), and the temperature varied between 18 and 22 °C.

### Data analysis

Statistical analyses and graphics were performed in R [102], using packages from the tidyverse for data manipulation and visualization [103]. The fixed effects of blue light and UV-B treatment on genotype were assessed using analysis of variance (ANOVA). Linear mixed-effects models were fitted using the lme4 package [104]. Growth compartment was treated as the unit of replication, with genotype nested within compartment as a random effect. Significance of fixed effects was evaluated using ANOVA tables with Satterthwaite’s approximation for degrees of freedom as implemented in the lmerTest package [105]. The contribution of the random effect of compartment was tested using likelihood ratio tests via the ranova function. Pairwise comparisons for significant interaction terms were conducted using estimated marginal means with the emmeans package [106], with *p*-values adjusted for multiple testing using the false discovery rate (FDR) method.

## Supplementary Information

Supplementary Material 1.

## Data Availability

The datasets generated and/or analysed during the current study are available at https:/doi.org/10.5281/zenodo.17544837(Zenodo record number 17544837).
