# Supplementary material for: Effects of blue light and UV-B radiation during growth on photochemical yields during exposure to bright sunlight
Source: BMC Plant Biol. 2026 Mar 6;26:672. doi: 10.1186/s12870-026-08455-4 (PMC13078021; doi:10.1186/s12870-026-08455-4)

**Table S1: Summary of statistical output from linear mixed model *for optical measurements of adaxial leaf flavonols, anthocyanins and chlorophyll; maximum quantum efficiency of PSII (F_V_/F_M_) and effective quantum yield of PSII (F_Q_/F_M_').*** *Values shown represent the p-values of the effect of the genotype (wild-type, phot_1_, cry_1_cry_2_, uvr_8-2_ and cry_1_cry_2_uvr_8-2_), the treatment (No UV-B No Blue, No UV-B full PAR, UV-B No Blue, UV-B full PAR), the days after germination (DAG 39 & 42 before and DAG 45 after highlight treatment); as well as the combined effect of Genotype and Treatment (G:T), Genotype and DAG (G:DAG), Treatment and Genotype (T:G), and the three way interaction of Genotype, Treatment , and DAG (G:T:DAG). P-values were adjusted for multiple comparisons using the false discovery rate (FDR) method. Random effect of the compartment in which the plants were positioned during growth was assessed using the RANOVA function available in the package lmerTEST (Kuznetsova et al., 2017). Signif. codes: 0 ‘***’ 0.001 ‘**’ 0.01 ‘*’ 0.05 ‘.’ 0.1 ‘ ’ 1*

| Trait | Genotype (G) | Treatment (T) | Days after germination (DAG) | G:T | G:DAG | T:G | G:T:DAG | Random effect  RANOVA |
| --- | --- | --- | --- | --- | --- | --- | --- | --- |
| Flavonoids index | < 0.001 *** | < 0.001 *** | < 0.001 *** | < 0.001 *** | 0.005 ** | < 0.001 *** | 0.091 | 0.008 ** |
| Anthocyanins index | < 0.001 *** | 0.026 * | < 0.001 *** | < 0.001 *** | < 0.001 *** | < 0.001 *** | < 0.001 *** | < 0.001 *** |
| Chlorophyll index | < 0.001 *** | 0.038 * | < 0.001 *** | < 0.001 *** | < 0.001 *** | < 0.001 *** | 0.431 | < 0.001 *** |
| F_V_/F_M_ | < 0.001 *** | 0.344 | < 0.001 *** | 0.001 ** | 0.002 ** | 0.095 | 0.118 | < 0.001 *** |
| F_Q_/F_M’_ | < 0.001 *** | 0.029 * | < 0.001 *** | < 0.001 *** | 0.221 | < 0.001 *** | 0.135 | < 0.001 *** |

**Table S2: Statistical output from linear mixed model for optical measurements of adaxial leaf flavonols***. Values shown represent the p-values of the effect of the genotype (wild-type, phot_1_, cry_1_cry_2_, uvr_8-2_ and cry_1_cry_2_uvr_8-2_), the treatment (No UV-B No Blue, No UV-B full PAR, UV-B No Blue, UV-B full PAR), the days after germination (DAG 39 & 42 before and DAG 45 after highlight treatment); as well as the combined effect of Genotype and Treatment, Genotype and DAG, Treatment and Genotype, and the three way interaction of Genotype, Treatment , and DAG. P-values were adjusted for multiple comparisons using the false discovery rate (FDR) method. Random effect of the compartment in which the plants were positioned during growth was assessed using the RANOVA function available in the package lmerTEST (Kuznetsova et al., 2017). Sum and mean squares (Sum Sq and Mean Sq, respectively) as well as numerator and denominator degrees of freedom (NumDF and DenDF, respectively) are shown. Signif. codes: 0 ‘***’ 0.001 ‘**’ 0.01 ‘*’ 0.05 ‘.’ 0.1 ‘ ’ 1*

|  | Sum Sq | Mean Sq | NumDF | DenDF | F value | P value | Significance |
| --- | --- | --- | --- | --- | --- | --- | --- |
| Genotype (G) | 3.285 | 0.821 | 4 | 579.169 | 63.867 | < 0.001 | *** |
| Treatment (T) | 1.961 | 0.654 | 3 | 7.737 | 50.839 | < 0.001 | *** |
| DAG | 3.006 | 1.503 | 2 | 575.595 | 116.870 | < 0.001 | *** |
| G:T | 3.762 | 0.314 | 12 | 575.457 | 24.383 | < 0.001 | *** |
| G:DAG | 0.284 | 0.036 | 8 | 574.880 | 2.764 | 0.005 | ** |
| T:DAG | 1.517 | 0.253 | 6 | 575.833 | 19.666 | < 0.001 | ** |
| G:T:DAG | 0.437 | 0.018 | 24 | 574.908 | 1.417 | 0.091 | . |

**Table S3: Statistical output from linear mixed model for optical measurements of adaxial leaf anthocyanins***. Values shown represent the p-values of the effect of the genotype (wild-type, phot_1_, cry_1_cry_2_, uvr_8-2_ and cry_1_cry_2_uvr_8-2_), the treatment (No UV-B No Blue, No UV-B full PAR, UV-B No Blue, UV-B full PAR), the days after germination (DAG 39 & 42 before and DAG 45 after highlight treatment); as well as the combined effect of Genotype and Treatment, Genotype and DAG, Treatment and Genotype, and the three way interaction of Genotype, Treatment , and DAG. P-values were adjusted for multiple comparisons using the false discovery rate (FDR) method. Random effect of the compartment in which the plants were positioned during growth was assessed using the RANOVA function available in the package lmerTEST (Kuznetsova et al., 2017). Sum and mean squares (Sum Sq and Mean Sq, respectively) as well as numerator and denominator degrees of freedom (NumDF and DenDF, respectively) are shown. Signif. codes: 0 ‘***’ 0.001 ‘**’ 0.01 ‘*’ 0.05 ‘.’ 0.1 ‘ ’ 1*

|  | Sum Sq | Mean Sq | NumDF | DenDF | F value | P value | Significance |
| --- | --- | --- | --- | --- | --- | --- | --- |
| Genotype (G) | 0.197 | 0.049 | 4 | 671.331 | 100.437 | < 0.001 | *** |
| Treatment (T) | 0.008 | 0.003 | 3 | 7.955 | 5.336 | 0.026 | * |
| DAG | 0.028 | 0.014 | 2 | 668.816 | 28.800 | < 0.001 | *** |
| G:T | 0.096 | 0.008 | 12 | 671.202 | 16.223 | < 0.001 | *** |
| G:DAG | 0.026 | 0.003 | 8 | 668.421 | 6.547 | < 0.001 | *** |
| T:DAG | 0.031 | 0.005 | 6 | 668.978 | 10.479 | < 0.001 | *** |
| G:T:DAG | 0.032 | 0.001 | 24 | 668.494 | 2.745 | < 0.001 | *** |

**Table S4: Statistical output from linear mixed model for optical measurements of adaxial leaf chlorophyll***. Values shown represent the p-values of the effect of the genotype (wild-type, phot_1_, cry_1_cry_2_, uvr_8-2_ and cry_1_cry_2_uvr_8-2_), the treatment (No UV-B No Blue, No UV-B full PAR, UV-B No Blue, UV-B full PAR), the days after germination (DAG 39 & 42 before and DAG 45 after highlight treatment); as well as the combined effect of Genotype and Treatment, Genotype and DAG, Treatment and Genotype, and the three way interaction of Genotype, Treatment , and DAG. P-values were adjusted for multiple comparisons using the false discovery rate (FDR) method. Random effect of the compartment in which the plants were positioned during growth was assessed using the RANOVA function available in the package lmerTEST (Kuznetsova et al., 2017). Sum and mean squares (Sum Sq and Mean Sq, respectively) as well as numerator and denominator degrees of freedom (NumDF and DenDF, respectively) are shown. Signif. codes: 0 ‘***’ 0.001 ‘**’ 0.01 ‘*’ 0.05 ‘.’ 0.1 ‘ ’ 1*

|  | Sum Sq | Mean Sq | NumDF | DenDF | F value | P value | Significance |
| --- | --- | --- | --- | --- | --- | --- | --- |
| Genotype (G) | 7492.576 | 1873.144 | 4 | 707.008 | 178.843 | < 0.001 | *** |
| Treatment (T) | 149.373 | 49.791 | 3 | 7.507 | 4.754 | 0.038 | * |
| DAG | 1578.863 | 789.432 | 2 | 703.596 | 75.373 | < 0.001 | *** |
| G:T | 3311.262 | 275.938 | 12 | 706.299 | 26.346 | < 0.001 | *** |
| G:DAG | 529.000 | 66.125 | 8 | 703.296 | 6.313 | < 0.001 | *** |
| T:DAG | 731.594 | 121.932 | 6 | 703.841 | 11.642 | < 0.001 | *** |
| G:T:DAG | 257.524 | 10.730 | 24 | 703.397 | 1.024 | 0.431 |  |

**Table S5: Spearman rank correlation coefficients among leaf pigment traits measured across genotypes.**

Correlations were calculated using Spearman’s rank correlation. P-values were adjusted for multiple comparisons using the false discovery rate (FDR) method.

| Treatment | Variable 1 | Variable 2 | Correlation | statistic | p-value | method | Adjusted p-value |
| --- | --- | --- | --- | --- | --- | --- | --- |
| No UV-B & Full PAR | Flavonoids | Anthocyanins | -0.6 | 534422.7 | < 0.001 | Spearman | < 0.001 |
| No UV-B & No Blue | Flavonoids | Anthocyanins | -0.47 | 1337820.2 | < 0.001 | Spearman | < 0.001 |
| UV-B & Full PAR | Flavonoids | Anthocyanins | -0.68 | 1103919.4 | < 0.001 | Spearman | < 0.001 |
| UV-B & No Blue | Flavonoids | Anthocyanins | 0.089 | 502433.4 | 0.282 | Spearman | 0.282 |
| No UV-B & Full PAR | Flavonoids | Chlorophyll | 0.63 | 124869.1 | < 0.001 | Spearman | < 0.001 |
| No UV-B & No Blue | Flavonoids | Chlorophyll | 0.28 | 698498.1 | < 0.001 | Spearman | < 0.001 |
| UV-B & Full PAR | Flavonoids | Chlorophyll | 0.62 | 243322.2 | < 0.001 | Spearman | < 0.001 |
| UV-B & No Blue | Flavonoids | Chlorophyll | 0.17 | 447565.8 | 0.037 | Spearman | 0.037 |
| No UV-B & Full PAR | Anthocyanins | Chlorophyll | -0.82 | 870335.9 | < 0.001 | Spearman | < 0.001 |
| No UV-B & No Blue | Anthocyanins | Chlorophyll | -0.61 | 3298347.1 | < 0.001 | Spearman | < 0.001 |
| UV-B & Full PAR | Anthocyanins | Chlorophyll | -0.9 | 1558527.4 | < 0.001 | Spearman | < 0.001 |
| UV-B & No Blue | Anthocyanins | Chlorophyll | -0.68 | 1021691.6 | < 0.001 | Spearman | < 0.001 |

**Table S6: Statistical output from linear mixed model for F_V_/F_M_ measurements by chlorophyll fluorescence***. Values shown represent the p-values of the effect of the genotype (wild-type, phot_1_, cry_1_cry_2_, uvr_8-2_ and cry_1_cry_2_uvr_8-2_), the treatment (No UV-B No Blue, No UV-B full PAR, UV-B No Blue, UV-B full PAR), the days after germination (DAG 39 & 42 before and DAG 45 after highlight treatment); as well as the combined effect of Genotype and Treatment, Genotype and DAG, Treatment and Genotype, and the three way interaction of Genotype, Treatment , and DAG. P-values were adjusted for multiple comparisons using the false discovery rate (FDR) method. Random effect of the compartment in which the plants were positioned during growth was assessed using the RANOVA function available in the package lmerTEST (Kuznetsova et al., 2017). Sum and mean squares (Sum Sq and Mean Sq, respectively) as well as numerator and denominator degrees of freedom (NumDF and DenDF, respectively) are shown. Signif. codes: 0 ‘***’ 0.001 ‘**’ 0.01 ‘*’ 0.05 ‘.’ 0.1 ‘ ’ 1*

|  | Sum Sq | Mean Sq | NumDF | DenDF | F value | P value | Significance |
| --- | --- | --- | --- | --- | --- | --- | --- |
| Genotype (G) | 0.544 | 0.136 | 4 | 289.121 | 15.131 | < 0.001 | *** |
| Treatment (T) | 0.033 | 0.011 | 3 | 11.421 | 1.228 | 0.344 |  |
| DAG | 3.945 | 1.315 | 3 | 28.850 | 146.376 | < 0.001 | *** |
| G:T | 0.651 | 0.054 | 12 | 288.856 | 6.041 | < 0.001 | *** |
| G:DAG | 0.291 | 0.024 | 12 | 286.880 | 2.698 | 0.002 | ** |
| T:DAG | 0.152 | 0.017 | 9 | 29.194 | 1.881 | 0.095 | . |
| G:T:DAG | 0.424 | 0.012 | 36 | 286.755 | 1.312 | 0.118 |  |

**Table S7: Statistical output from linear mixed model for F_Q_/F_M’_ measurements by chlorophyll fluorescence***. Values shown represent the p-values of the effect of the genotype (wild-type, phot_1_, cry_1_cry_2_, uvr_8-2_ and cry_1_cry_2_uvr_8-2_), the treatment (No UV-B No Blue, No UV-B full PAR, UV-B No Blue, UV-B full PAR), the days after germination (DAG 39 & 42 before and DAG 45 after highlight treatment); as well as the combined effect of Genotype and Treatment, Genotype and DAG, Treatment and Genotype, and the three way interaction of Genotype, Treatment , and DAG. P-values were adjusted for multiple comparisons using the false discovery rate (FDR) method. Random effect of the compartment in which the plants were positioned during growth was assessed using the RANOVA function available in the package lmerTEST (Kuznetsova et al., 2017). Sum and mean squares (Sum Sq and Mean Sq, respectively) as well as numerator and denominator degrees of freedom (NumDF and DenDF, respectively) are shown. Signif. codes: 0 ‘***’ 0.001 ‘**’ 0.01 ‘*’ 0.05 ‘.’ 0.1 ‘ ’ 1*

|  | Sum Sq | Mean Sq | NumDF | DenDF | F value | P value | Significance |
| --- | --- | --- | --- | --- | --- | --- | --- |
| Genotype (G) | 0.506 | 0.127 | 4 | 769.834 | 14.809 | < 0.001 | *** |
| Treatment (T) | 0.111 | 0.037 | 3 | 11.215 | 4.347 | 0.029 | * |
| DAG | 8.697 | 2.899 | 3 | 36.697 | 339.223 | < 0.001 | *** |
| G:T | 0.689 | 0.057 | 12 | 767.213 | 6.720 | < 0.001 | *** |
| G:DAG | 0.132 | 0.011 | 12 | 765.456 | 1.286 | 0.221 |  |
| T:DAG | 0.314 | 0.035 | 9 | 37.592 | 4.082 | 0.001 | ** |
| G:T:DAG | 0.391 | 0.011 | 36 | 764.734 | 1.271 | 0.135 |  |

**Fig. S1. Maximum fluorescence measured by chlorophyll fluorescence on light adapted leaves of *Arabidopsis* wild-type and photoreceptor mutants** (see Methods), growing under different light treatment and moved to natural sunlight outdoors. Data before the move outdoors was pooled, and all genotypes are shown together. Values shown represent the mean ± 1 standard error (SE), calculated from plants grown in three replicate compartments for each treatment.


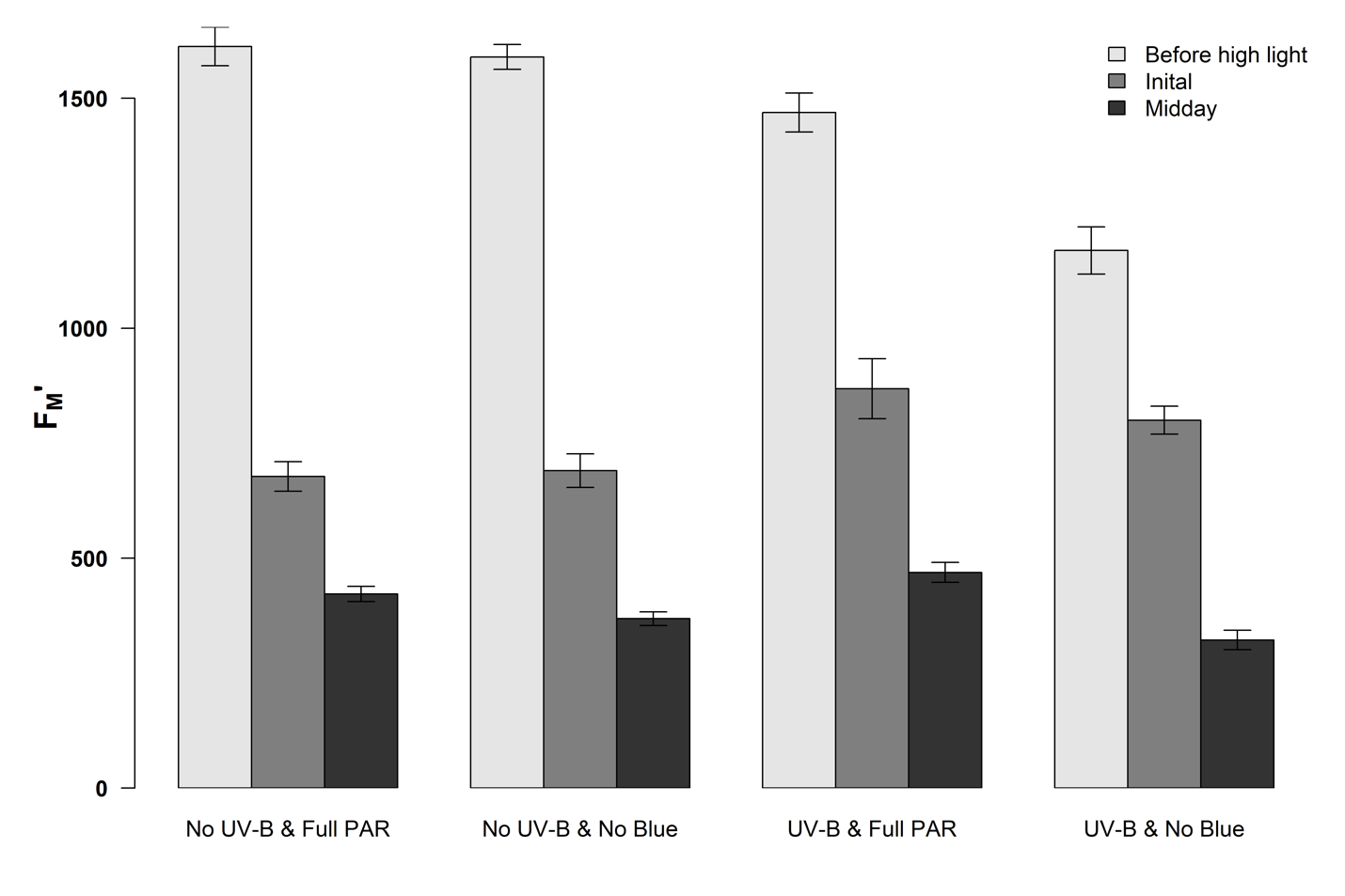

Supplement: Supplementary file 1 — Supplementary Material 1. [file 12870_2026_8455_MOESM1_ESM.docx]
